# Effects of rearing mode on gastro-intestinal microbiota and development, immunocompetence, sanitary status and growth performance of lambs from birth to two months of age

**DOI:** 10.1186/s42523-023-00255-7

**Published:** 2023-07-17

**Authors:** Lysiane Dunière, Philippe Ruiz, Yacine Lebbaoui, Laurie Guillot, Mickael Bernard, Evelyne Forano, Frédérique Chaucheyras-Durand

**Affiliations:** 1 Lallemand SAS, CEDEX, 19 rue des Briquetiers, BP 59, Blagnac, 31702 France; 2 Université Clermont Auvergne, INRAE, UMR 454 MEDIS (Microbiologie Environnement Digestif et Santé), Clermont-Ferrand, 63000 France; 3 UE 1414 (Unité Expérimentale), INRAE, Herbipôle, Saint-Genès Champanelle, 63122 France

**Keywords:** Microbiota, Rumen, Feces, Artificial rearing, Lambs, IgG, ADG

## Abstract

**Background:**

Artificial rearing system, commonly used in prolific sheep breeds, is associated to increased mortality and morbidity rates before weaning, which might be linked to perturbations in digestive tract maturation, including microbiota colonization. This study evaluated the effect of rearing mode (mothered or artificially reared) on the establishment of the rumen and intestinal microbiome of lambs from birth to weaning. We also measured immunological and zootechnical parameters to assess lambs’ growth and health. GIT anatomy as well as rumen and intestinal epithelium gene expression were also analysed on weaned animals to assess possible long-term effects of the rearing practice.

**Results:**

Total VFA concentrations were higher in mothered lambs at 2 months of age, while artificially-reared lambs had lower average daily gain, a more degraded sanitary status and lower serum IgG concentration in the early growth phase. Metataxonomic analysis revealed higher richness of bacterial and eukaryote populations in mothered vs. artificially-reared lambs in both Rumen and Feces. Beta diversity analysis indicated an evolution of rumen and fecal bacterial communities in mothered lambs with age, not observed in artificially-reared lambs. Important functional microorganisms such as the cellulolytic bacterium *Fibrobacter succinogenes* and rumen protozoa did not establish correctly before weaning in artificially-reared lambs. Enterobacteriaceae and *Escherichia coli* were dominant in the fecal microbiota of mothered lambs, but main *E. coli* virulence genes were not found differential between the two groups, suggesting they are commensal bacteria which could exert a protective effect against pathogens. The fecal microbiota of artificially-reared lambs had a high proportion of lactic acid bacteria taxa. No difference was observed in mucosa gene expression in the two lamb groups after weaning.

**Conclusions:**

The rearing mode influences gastrointestinal microbiota and health-associated parameters in offspring in early life: rumen maturation was impaired in artificially-reared lambs which also presented altered sanitary status and higher risk of gut dysbiosis. The first month of age is thus a critical period where the gastrointestinal tract environment and microbiota are particularly unstable and special care should be taken in the management of artificially fed newborn ruminants.

**Supplementary Information:**

The online version contains supplementary material available at 10.1186/s42523-023-00255-7.

## Background

The microbial colonization of the digestive tract during early life is a dynamic process in mammals which plays a key role in host health and development [1, 2]. Indeed, gastro-intestinal tract (GIT) microbiota impacts the host immune system development and gut maturity, in addition to an important role in feed/food digestion and fermentation as well as preventing from pathogen infections. In ruminants, weaning is a critical period during which the digestive system switches from that of a monogastric hindgut fermenter to that of a foregut fermenter, with the development of the rumen. The mature rumen houses a very dense and diverse microbiota, composed of bacteria, archaea, protozoa and fungi which are responsible for the degradation and fermentation of the diet ingested by the animal. Even if the newborn animal is exclusively fed with milk during the first days or weeks and that milk bypasses the rumen due to the oesophageal groove closure, it has been known for a long time that the rumen is colonized by a diversified microbiota very early after birth, and that the rumen is functional long before weaning [3–8].

The rumen microbiota is inoculated at birth and in early life from various sources, including the mother vaginal canal, udder skin, colostrum but also environment, fecal material on the litter and saliva from the dam [9]. It has been shown that the presence of the dam or of other adult ruminants is important for cellulolytic bacteria, fungi and protozoa colonization in the neonate [3, 8]. In addition, newborns separated from their dam after birth had an impaired establishment of the cellulolytic bacterium *Fibrobacter succinogenes* and of protozoa in their rumen [10]. Furthermore, maternal deprivation produces detrimental psychological effects on young animals [11] in addition to a possible incomplete transfer of immunity.

Although the presence of the dam is of importance for the newborn gut microbiota inoculation and its health and welfare, artificial rearing systems, i.e., separating newborns from their mothers and feeding them with milk replacer, have been developed in ovine. This is due to the selection of prolific breeds such as Romane breed with ewes giving birth up to 3 or even 4 lambs, because of the important economic impact of reproductive efficiency in sheep, particularly for meat production [12]. However, large litter ewes do not have enough milk to nurse all their lambs until weaning, which leads to the development of artificial rearing systems. These rearing practices induce animal stress and impair early growth rate, health and emotional state mainly during the milk-feeding period and at weaning [12]. Maternal deprivation represents also a nutritional challenge due to artificial feeding since the milk replacer contains various non-dairy products in addition to whey and low-fat milk powder, which may lead to poorer growing performance [12]. In artificial rearing systems, lambs are usually left with their mothers at least one day after birth before separation, to allow them to take maternal colostrum and the included immunoglobulins (Ig) [13, 14]. Inadequate absorption of colostral Igs through the gut has been shown to increase neonatal mortality in lambs [15–17], and artificial rearing could be associated with failure of passive immunity transfer [18, 19].

Rumen development could also be negatively affected by artificial rearing, and kids isolated and fed with milk replacer could also show a significant growth impairment [20]. However, these effects have not been always observed [21]. The negative effect on rumen development could be due to differences in microbes’ colonization that were observed in artificially-reared animals [19, 20, 22, 23]. It has been suggested that, in addition to strong deterministic constraints imposed by diet and age, stochastic colonization in early life could have long-lasting impacts on the development of animal microbiomes [21, 24]. Then, the objective of the present work was to assess the effect of rearing mode on microbial, immunological and zootechnical parameters of lambs. We hypothesized that nutritional interventions in early life such as different milk-feeding conditions could have immediate effects on the animal’s health, performance and GIT microbiota colonization, with some effects possibly persistent after weaning. We compared the microbial colonization and development processes of the GIT in lambs subjected to two rearing systems: lambs left with their mother and lambs separated from their dam and fed milk replacer. We used a metataxonomic analysis of bacteria and eukaryotes to monitor the evolution of the rumen and fecal microbiome from birth to weaning and measured immunological and zootechnical parameters to assess lambs’ growth and health. GIT anatomy as well as rumen and intestinal epithelium gene expression were quantified on weaned animals to assess possible long-term effects of the rearing practice on GIT development and mucosa metabolism, respectively.

## Results

### Animal performances and sanitary status

**Fig. 1:**
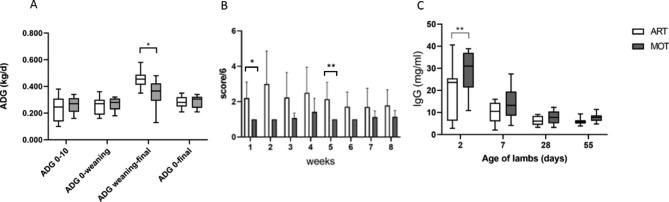
Performances and sanitary status of the mothered (MOT, grey box) and artificially reared (ART, white box) lambs. Average daily gain (ADG) **(A)**, welfare score **(B)** and serum IgG concentration **(C)** of the two groups of lambs. **B**: Mixed-effects model (REML) showed an effect of rearing mode (p < 0.001) on welfare score. The differences in the two groups were found significant at week 1 (p < 0.05) and 5 (p < 0.005) (Bonferoni multiple comparison test). **C**: Mixed-effects model (REML) showed effect of age (p < 0.0001), rearing mode (p < 0.05) and interaction between them (p < 0.05) on IgG concentration in serum of lambs. Sidak’s multiple comparison test indicated that MOT IgG were higher than ART IgG at day 2 (p = 0.005)

Lamb growth was monitored all along the experiment. On the whole experiment, no significant difference was observed in average daily gain (ADG) of MOT and ART groups, although the ADG was significantly higher for the ART group in the post-weaning period (Fig. 1A) (*p* < 0.05). In addition, the MOT group showed higher variability in ADG after weaning.

The sanitary status of the lambs was monitored using a welfare score [12] (Fig. 1B). A more degraded welfare score was observed in ART lambs at weeks 1 and 5, mainly due to the dirtiness of the rear end.

The lamb immunological status was assessed by quantification of blood serum IgG. IgG concentration was strongly affected by the rearing mode during the first days of life, suggesting a limited immune passive transfer in ART compared to MOT lambs (Fig. 1C). Indeed, 3 lambs from the ART group had IgG concentration < 10 g/l, which could be qualified as failure of passive transfer (FPT). No death was recorded along the experimental period.

### Rumen pH and SCFA

The pH and the SCFA concentrations were measured on rumen samples taken by oesophageal tubing on all the lambs. The ruminal pH was highly variable in ART lambs during the 1st week (from 5.54 to 7.59; Supplementary Fig S1), whereas MOT rumen pH appeared more stable and was significantly higher than ART rumen pH at day 28 (p < 0.05), with an effect of Time x Rearing mode interaction (p < 0.01).

Total SCFA concentrations were higher in MOT lambs at day 55(56.86 vs. 28.27 mM in MOT and ART groups respectively, p < 0.01) (Supplementary Fig S2). Accordingly, the acetate, propionate, butyrate and valerate concentrations were significantly higher in MOT lamb rumens at day 55. In addition, the isovalerate and isobutyrate concentrations were higher in MOT lamb rumens at day 7 (Supplementary Fig S2). The analysis of the relative proportions of SCFA in the two groups showed lower proportions of acetate at day 28 but higher proportions of butyrate at days 28 and 55 in MOT rumens (Table 1). Higher proportions of isovalerate and isobutyrate were also calculated at day 7 in the rumen of MOT lambs.

**Table 1:** Relative proportions of SCFA in the two lamb groups

	% Acetate		% Propionate		% Butyrate		% isobutyrate		%isovalerate		% valerate	
days	ART	MOT	ART	MOT	ART	MOT	ART	MOT	ART	MOT	ART	MOT
3	76.8	71.4	18.5	22.1	3.13	3.33	0.70	1.59	0.50	1.38	0.27	0.16
5	80.7	74.3	16.0	18.5	2.07	3.78	0.63	1.63	0.38	1.60	0.19	0.22
7	77.2	68.7	17.2	22.4	3.92	3.53	**0.85a**	**2.26b**	**0.59a**	**2.39b**	0.21	0.53
14	74.6	76.7	18.0	16.2	2.92	3.86	1.85	1.17	2.04	1.35	0.55	0.57
28	**75.5a**	**67.5b**	17.2	19.2	**2.07a**	**6.64b**	2.01	2.32	2.39	3.06	0.82	1.15
55	59.1	58.1	25.5	21.3	**9.53c**	**15.50d**	1.66	0.82	1.78	0.90	2.12	2.62

Post-hoc tests: significant differences are in bold. Acetate: *p* = 0.0065 (a,b); butyrate: *p* = 0.0303 (a,b), *p* = 0.0076 (c,d); isobutyrate: *p* = 0.0002 (a,b); isovalerate: *p* = 0.0009 (a,b)

### Establishment of the rumen and intestinal microbiota in MOT and ART lambs

We used a metataxonomic approach together with DNA-based quantification (quantitative PCR) of microbiota to compare the rumen microbial colonization of mothered and artificially-reared lambs from birth up to two months of life and monitored the evolution of the fecal microbiota of the two groups during the same period of time.

### Establishment of the rumen microbiota

#### Quantification of targeted bacteria by qPCR

**Fig. 2:**
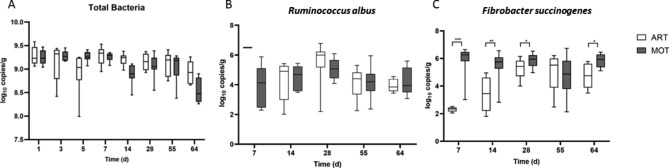
qPCR quantification of bacterial populations in the rumen of ART and MOT lambs Targeted populations were total bacteria **(A)**, *Ruminococcus albus***(B)** and *Fibrobacter succinogenes***(C)**. The primer sequences are given in material and methods section. White box: ART lambs; grey boxes: MOT lambs

Total bacteria and two major cellulolytic bacterial species, *Ruminococcus albus* and *Fibrobacter succinogenes* were quantified in the rumen of all animals from the two groups using qPCR (Fig. 2). Total bacteria were quantified at high levels from the first days of life (~ 9 Log_10_ of 16 S rRNA gene copy numbers/g), but no significant difference was found between ART and MOT lambs, and a high variability was observed, especially during the first 7 days of life. No difference could be detected on *R. albus* population levels in the two groups, but *F. succinogenes* established more rapidly at high levels in the MOT lambs compared to the ART lambs (Fig. 2).

#### Effect of rearing mode on the rumen bacteria composition and diversity

The rumen microbiota of 8 animals from each of the two lamb groups was analysed using 16 S rRNA gene amplicon sequencing on samples taken at 7, 14 and 28 days of age (pre-weaning).

**Fig. 3:**
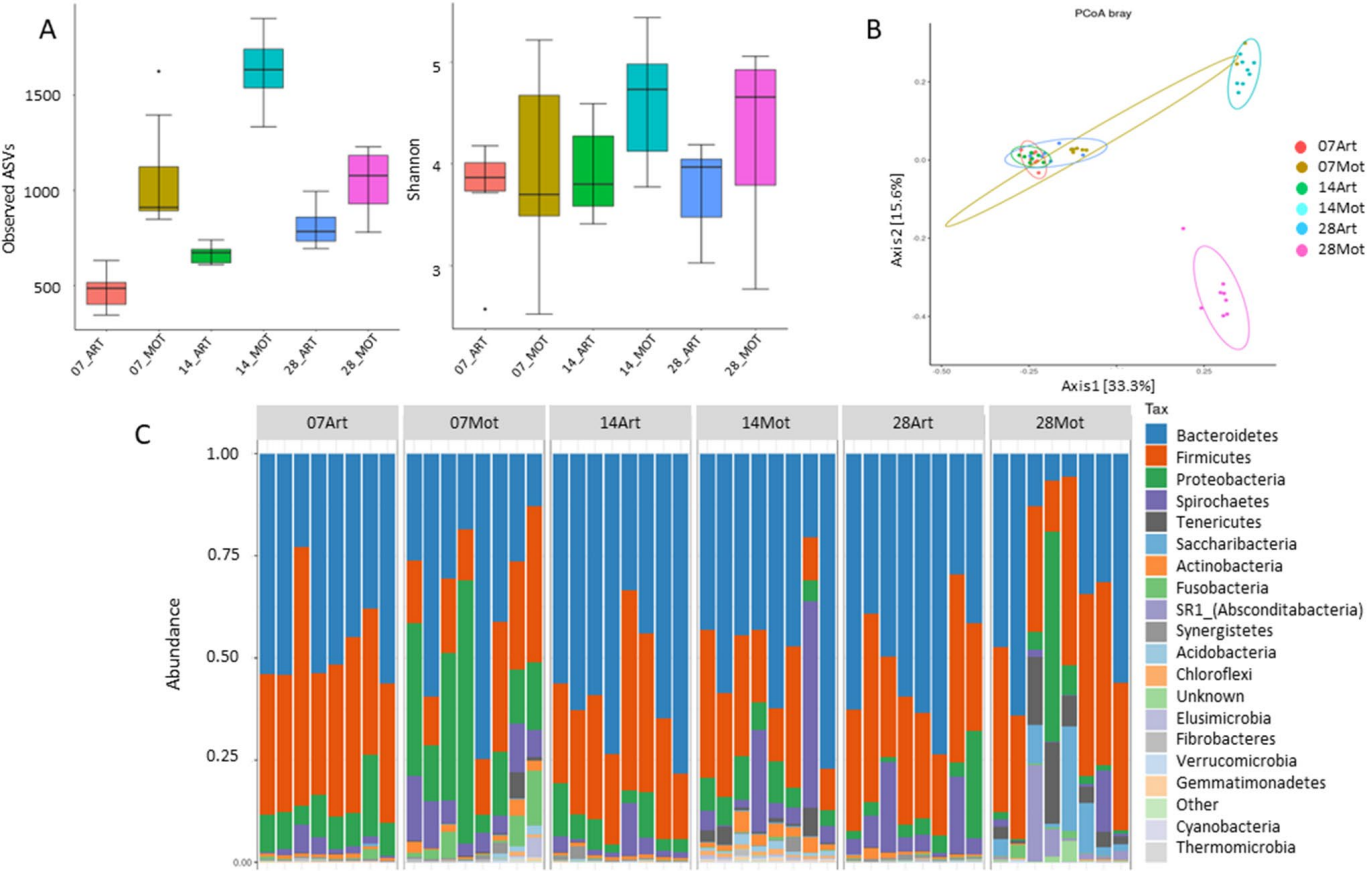
Alpha and beta diversity of the rumen bacteria in MOT and ART lambs **A**: alpha diversity analysis (Observed ASV and Chao1 index) of ART and MOT lamb rumen bacteria; **B**: PCoA plot based on Bray–Curtis distance for normalized abundance data; **C**: Barplots of bacterial ASVs by phylum (individual sample composition)

A group effect was observed for ASV numbers and Shannon index with higher values in the rumen microbiota of mothered lambs compared with artificially-reared lambs (Fig. 3A, p < 0.01), indicating higher richness in the MOT rumens whatever the sampling time considered. A global time effect was also observed for bacterial richness (p < 0.01, Fig. 3A).

Beta diversity analysis of the rumen microbiota showed an evolution of bacterial communities with age in MOT lambs not observed in ART lambs (Fig. 3B). A significant effect of group was observed (p < 0.01) as well as interaction between time and group (p < 0.01).

At the phylum level, MOT lamb rumens had much more relative proportions of Proteobacteria at day 7 than ART lambs (on average 25.16% vs. 10.76% in MOT and ART groups respectively, Fig. 3C). In addition, Tenericutes were present in several MOT lambs from day 14 up to day 28, and Actinobacteria and Spirochaetes proportions appeared also higher in the MOT rumens at 7 and 14 days for several animals, indicating a diversification of the rumen microbiota with age. On the contrary, in ART lamb rumens, Bacteroidetes, Firmicutes and Proteobacteria remained the main phyla whatever the day of sampling. Analysis at the family level indicated that Neisseriaceae represented an important proportion of the Proteobacteria found in MOT rumens, while the proteobacteria in ART rumens were mostly Enterobacteriaceae (Supplementary Fig S3). Prevotellaceae was the major family of microbiota from ART animals, while the microbiota of MOT lambs appeared more diversified also at the family level.

Differential analysis using Deseq2 identified many differential taxa between the two lamb groups, whatever the day of sampling (p < 0.05). Twenty-four differential taxa were identified at day 7, 120 differential taxa at day 14 and 51 at day 28 (Supplementary Material S1.xlsx). At day 7, only 5 taxa were more abundant in ART rumens while 19 other taxa were more abundant in MOT rumens. Differential taxa at day 7 included Lactobacillaceae, Enterobacteriaceae, Streptococcaceae and Veillonellaceae which were more abundant in ART rumens (Log2FC > 2.5) and taxa such as Acidobacteriaceae (subgroup 1), Bradyrhizobiaceae, Anaeroplasmataceae or Neisseriaceae more abundant in MOT rumens (Log2FC<-3.5). Fibrobacteraceae were also much more abundant in MOT rumens (Log2FC<-2).

The differential analysis also identified differential taxa in relation to age (p < 0.05), these differential taxa being much more numerous in the MOT group compared with the ART group. The number of differential families between day 7 and 14 of age were 10 and 44 for ART and MOT rumens, respectively, and between 14 and 28 days, the differential families were 23 for ART and 114 for MOT.

The observations made through DESeq2 analysis were confirmed by the indicspecies analysis (Supplementary Table S1) : Among species with relative abundance above 1%, *Lactobacillus* (*L. ingluviei* and *L. saerimneri*, 1.05% total) and *E.coli* (5.2%) were characterized as indicative of Art group at d7 (p < 0.05). Some *Streptococcus* species were significantly associated with Art group (d7 and d14) but at low relative abundance (~ 0.05%). *Anaeroplasma varium* (1.5%) was indicative of Mot group at d28 while several *Acidobacteria* species were identified as significantly indicative of Mot group at d14 but at low relative abundances (ranging from 0.003 to 0.09%).

#### Effect of rearing mode on the rumen eukaryotic community

The rumen Eukaryota community was analysed using 18 S rRNA gene amplicon sequencing.

**Fig. 4:**
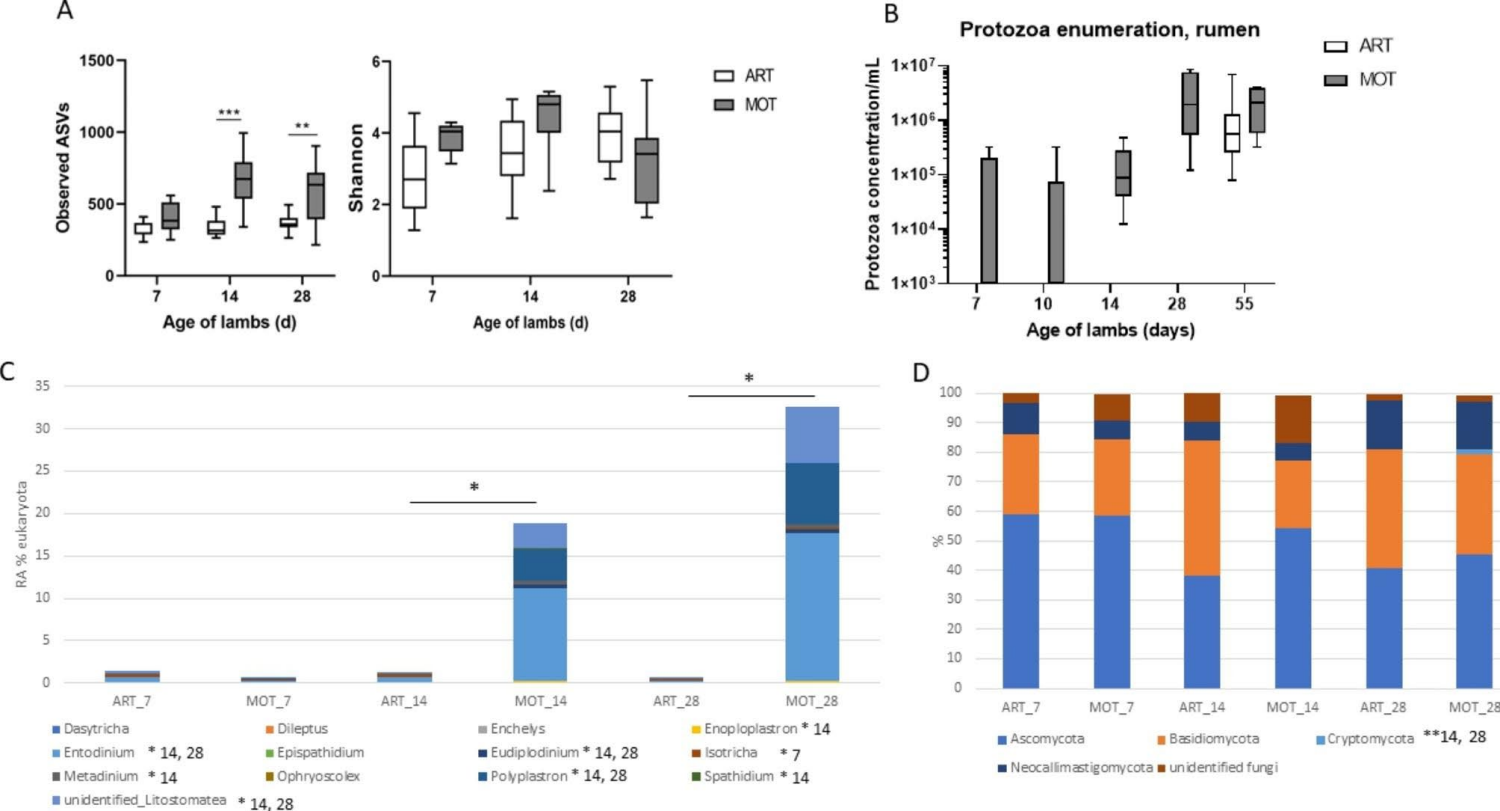
Effect of rearing mode on rumen eukaryota **A**: alpha diversity (observed OTUs and Chao 1 index) in rumens of ART (white box) and MOT (grey bow) lambs; **B**: protozoa enumeration by counting in the rumen samples from the two groups; **C**, **D**: composition of the protozoa **(C)** and fungi **(D)** communities in the rumens of the two groups

Eukaryota OTU numbers and Shannon index were found higher (p < 0.05) in the rumen microbiota of mothered lambs compared with artificially-reared lambs (Fig. 4A), indicating higher richness in the MOT rumens. Protozoa and fungi colonization were then analysed separately.

Rumen ciliate protozoa were enumerated by counting and were not detected before day 55 in ART rumens, while they were identified as soon as day 7 in mothered lambs (Fig. 4B). All of the MOT rumens were colonized by protozoa at day 14 while all of the ART rumens were colonized by protozoa only at day 55. The protozoal population grew in size and diversity with age in the MOT lamb rumens and appeared stabilized at 28 days. Metataxonomic analysis confirmed a significant effect of the rearing mode from day 14, with earlier rumen colonization of MOT lambs by total Litostomatea and by *Entodinium*, *Eudiplodinium* and *Polyplastron* genera, which were also the dominant taxa in these animals, while in ART lambs the dominant genera were *Isotricha* and *Dasytricha* (Fig. 4C). The rearing mode had also a significant effect on the presence of parasites, relative abundance of total parasite taxa and *Eimeria* at day 28 being higher in ART rumens compared with MOT rumens (Supplementary Fig S4).

Within fungi, identified taxa belonged to Ascomycota, Basidiomycota, Cryptomycota, Neocallimastigomycota and unidentified phyla (Fig. 4D). The relative abundance of Cryptomycota phylum was significantly higher in MOT group at day 14 and 28 (p < 0.01). *Cyllamyces*, *Orpinomyces* and Neocallimastigales were the major taxa identified in both MOT and ART rumens (Supplementary Fig S5A). Several taxa from the Saccharomycetales order were found with a higher relative abundance in ART rumens at day 14 (*Pichia*, *Candida*, *Kluyveromyces*, unidentified Saccharomycetales) and day 28 (*Pichia*, unidentified Saccharomycetales), except *Debaryomyces* sequences which were more abundant in MOT lamb rumens at day 14 (Supplementary Fig S5B).

### Evolution of the fecal microbiota

#### Quantification of targeted bacteria by qPCR

Total bacteria and targeted bacterial species or groups in the feces of all the ART and MOT lambs were quantified from 14 to 64 days (Supplementary Fig S6). A significant impact of the rearing mode was observed at day 14 for lactic acid bacteria (LAB) and at day 14 and 28 for Bifidobacteria, these bacterial populations being quantified at higher levels in ART lambs.

#### Effect of rearing mode on the fecal bacteria taxa and diversity and E. coli virulence genes

The bacterial taxonomic composition of the fecal microbiota was analysed on feces collected at 14 and 28 days from the same 8 animals from each of the MOT and ART groups. Regarding alpha diversity, ASV numbers and Shannon index were not found significantly different between the ART and MOT fecal microbiota (Supplementary Fig S7).

**Fig. 5:**
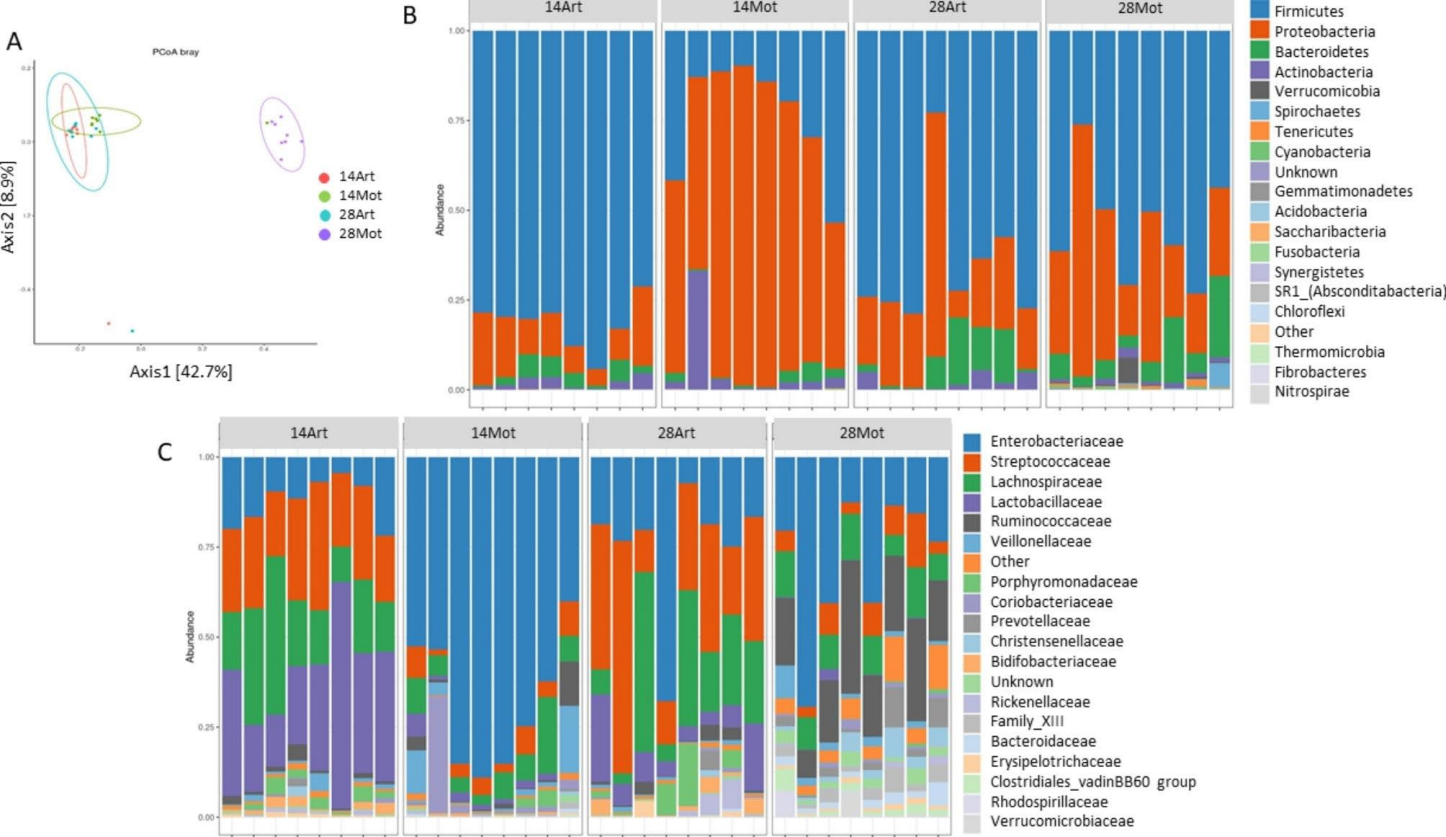
Alpha and beta diversity of the fecal bacteria in MOT and ART lambs **A**: PCoA plot based on Bray–Curtis distance for normalized abundance data; **B**, **C**: Barplots of bacterial ASVs by phylum **(B)** and family **(C)** (individual sample composition)

Beta-diversity analysis indicated, as for the rumen, an evolution of the fecal microbiota with age in MOT lambs, but not in ART lambs (p < 0.01, Fig. 5A).

The bacterial composition was clearly divergent between ART and MOT fecal samples, both at phylum and family levels (Fig. 5B and C). Indeed, at day 14, Proteobacteria was clearly the dominant phylum in the feces of 7 lambs out of 8 MOT animals, and these taxa belonged mostly to the Enterobacteriaceae family (Fig. 5B) and to *Escherichia* genus. In the ART fecal samples, Firmicutes was the dominant phylum, composed in majority of Streptococcaceae, Lachnospiraceae and Lactobacillaceae families, with *Streptococcus* and *Lactobacillus* as dominant genera within these families. At day 28, the microbiota of MOT samples was more diversified at the family and genus levels compared with day 14, and Enterobacteriaceae proportion decreased while Ruminococcaceae represented an important proportion of the detected families. Main difference between day 14 and 28 samples in the ART group was a decrease in Lactobacillaceae proportions with age. *Streptococcus* was a major genus found in ART feces at day 28.

Deseq2 analysis (Supplementary Material S2.xlsx) identified 10 differential taxa between MOT and ART feces (p < 0.05) at day 14: Lactobacillaceae, Alcaligenaceae, Streptococcaceae, Gemmatimonadaceae and Erysipelotrichaceae families were more abundant in ART feces, while Bacteroidaceae, Coriobacteriaceae, Enterobacteriaceae, Moraxellaceae and Pseudomonadaceae were more abundant in MOT feces. At day 28, 19 differential taxa were identified between ART and MOT fecal microbiota, with high value of Log2FC. These included Bifidobacteriaceae and Lactobacillaceae much more abundant in ART feces. Deseq2 analysis also identified 27 taxa differentially distributed according to age in MOT fecal samples (p < 0.05). Two species of lactic acid bacteria (*Pedicoccus acidilactici* 2.6% and *Lactobacillus ingluviei* 6.6%) were identified as significantly indicative of Art group at d14, while Mot group was associated with *E. coli* (67.1%, p < 0.05; Supplementary Table S1). Interestingly, *Bifidobacterium* sp. PG12A was associated with Art group at d28, strengthening both qPCR and DESeq2 analyses. Mot group at d28 was significantly associated with *Ruminococcus flavefaciens* (1.3%), *Faecalibacterium prausnitzii* (0.044%) and *Prevotella* HUN102 (0.4%).

Due to the highest proportion of *Escherichia* genus in MOT lambs, a focus was made to quantify selected genes associated to *E. coli* virulence. Sporadic detection of virulence genes was observed in fecal samples over time. Results were thus analysed as detection frequencies and not quantification. Among the 7 *E. coli* virulence genes tested, only 2 presented significant differences in detection frequencies between MOT and ART groups at d14 or d28. Gene encoding heat stable enterotoxin A (Sta) was more frequently detected in ART feces at d14 (11/13 vs. 0/11 animals in ART and MOT group respectively, p < 0.0001) while gene encoding Eagg EC heat stable enterotoxin (East1) was more prevalent in MOT feces at d14 (5/11 vs. 0/13 animals in MOT vs. ART group respectively, p = 0.011). The heat labile enterotoxin (LT), East1 and Shiga toxin 2 (Stx2) genes were present in some samples at d14 but not detected anymore at d28 in any sample, while Stx1 was below detection threshold in all fecal samples tested at both sampling times (Supplementary Table S2).

#### Effect of rearing mode on the fecal eukaryotic community

As for the rumen, Eukaryota OTU numbers index was found higher (p < 0.05) in the fecal microbiota of mothered lambs compared with artificially-reared lambs at day 28 (Supplementary Fig S8A), indicating higher richness in the MOT feces although Shannon index was not significantly different between groups.

Regarding fungi, Cryptomycota were more abundant in MOT fecal microbiota at day 14 and 28 (p < 0.05), and Chytridiomycota were more abundant in MOT feces at day 28 (p < 0.001) (Supplementary Fig S8B). As for the rumen, Saccharomycetales order was found with a higher relative abundance in ART samples at day 14 and 28, and within this order, *Wickerhamomyces* were higher in ART feces at day 28 (p < 0.05) (Supplementary Fig S8C). The rearing mode had a significant effect on the presence of parasite taxa in the feces, relative abundance of *Cryptosporidium* at day 28 being higher in ART fecal samples compared with MOT (p < 0.001) (Supplementary Fig S8D).

### Impact of the rearing mode on the gastrointestinal tract physiology

Five animals from each group were slaughtered at day 64, and GIT pH and anatomy, rumen papillae development, and intestinal gene expression were analysed.

*Organ pH and weight.* The pH in rumen, abomasum, small intestine, caecum, and colon contents was measured quickly after slaughter and organ collection. The pH was not found significantly different between the two groups (Supplementary Fig S9A). The weight of the various GIT organs was determined and reported relatively to the total GIT weight (Supplementary Fig S9B). No significant difference was observed between ART and MOT lambs at day 64. A numerically higher proportion of forestomach (29.75% in ART vs. 26.50% in MOT groups respectively, p = 0.248) and in parallel a lower proportion of lower gut (61.69% in ART vs. 66.73% in MOT groups respectively, p = 0.062) were observed across the GIT in ART lambs. Rumen wall thickness and papillae dimensions were also measured for the two groups, but no significant effect of the rearing mode was observed (Supplementary Table S3).

*Expression of targeted genes in rumen and colon epithelium.* Expression of genes coding for immune function, metabolic activity and epithelium integrity were quantified in rumen and colon epithelium sampled from the ART and MOT lambs at day 64 (Supplementary Fig S10). All the targeted genes were expressed in the rumen and colon epithelium, except TLR2 and TNFα for which no expression could be detected. Whatever the housekeeping gene used for relative expression quantification, no significant difference in expression of the selected genes was observed between epithelial cells of the ART and MOT groups. However, differences were observed in expression of these genes depending on the GIT location (Supplementary Fig S10). Whatever the housekeeping gene used and in both the ART and MOT groups, the genes encoding Claudin1 (tight junctions), HMGSC2 (3-hydroxy-3-methylglutaryl-CoA synthase isoform 2), HMGSL (3-hydroxy-3-methylglutaryl-CoA lyase) and MCT1 (monocarboxylate transporter 1) were more expressed in the rumen than in the colon epithelium (p < 0.05). On the contrary, the IGF-1 (insulin-like growth factor-1), IL-10 (interleukin 10 cytokine) and TLR4 (Toll like receptor 4) encoding genes were more expressed in the colon than in the rumen epithelial cells (p < 0.05). *IGF1* and *MCT1* were the most expressed genes in the two GIT locations.

## Discussion

This study was undertaken to investigate the impact of artificial rearing of lambs (i.e. separation from the dam and milk replacer feeding) on rumen and lower gut microbiota colonization in the first month after birth as well as possible post-weaning effects on GIT development.

### Performance and sanitary status of mothered and artificially-reared lambs

No difference in lambs’ weight and ADG was observed between groups before weaning or on the whole experiment. Several previous works reported impaired early growth rate in lambs submitted to artificial rearing compared with mothering [12, 22]. On the contrary, in the present study, the post-weaning ADG was significantly higher for the ART group, and appeared more variable in the MOT group, also after weaning. This higher variability in MOT lambs might result from the stress due to separation of lamb and dam at weaning. The composition of milk replacer varies from one supplier to another and could explain differences observed between studies, as well as the mode of suckling which differed also between studies (commercial buckets vs. in-house system used here). Our results suggest that the milk replacer and the suckling system we used allowed to cover well the lambs’ needs and avoided competition for teat allocation. One hypothesis to explain the higher ADG measured in the ART group after weaning could be that although MOT and ART lambs had access to solid feed at the same age (i.e. 8d), MOT lambs were not prone to rapidly shift from dam’s milk to feed. Another explanation could be higher nutrients mobilization in MOT lambs toward digestive tract development and immune system maturation and less toward growth, although no difference in expression of selected epithelium genes was found. ART lambs were likely to perceive digestive discomfort due to high milk replacer intake and thus tended to start consuming starch-rich pellets earlier in age before weaning. Indeed, a more degraded sanitary status was observed in ART lambs compared to MOT animals, at weeks 1 and 5. This was mainly due to dirtier perianal areas, indicating altered digestive transit. Previous work on lambs also showed minor diarrheal events from weeks 2 to 5 with artificial rearing, leading to lower growth rates during the 1st month but the animals reached similar weaning weights than mothered lambs [22], as we observed in the present study. The dirtiness of the rear end observed on ART lambs in the present work was associated to a disturbed microbiota in their feces, and detection of higher relative proportions of *Cryptosporidium* taxa. *Cryptosporidium* is an ubiquitous protozoan parasite that infects a broad range of vertebrate hosts, including sheep [25]. Note that total parasite taxa and *Eimeria*, a well-known coccidian parasite in lambs [26], were also found in higher proportions in ART than in MOT rumens. In addition to a lower sanitary score in the ART lambs, the IgG concentrations in blood serum measured on this group were lower than that measured in the MOT group in the first days of life, although the mean IgG concentrations were rather comparable to published literature (from 15.7 to 65 g/l) [22, 27]. In addition, 3 lambs from the ART group had a serum IgG level lower than 10 g/L, indicating failure of passive transfer (FPT). These results reflect a lower immunocompetence in the ART group at early age, probably due to the limited intake of colostrum during the 12 h lambs were kept with their mother. Colostrum ingestion is of paramount importance for the neonate and FPT is associated with an increased neonatal mortality [28]. Delaying colostrum feeding within 12 h of life has been shown to decrease the passive transfer of IgG in calves [29]. In agreement with these observations, lower serum IgG has been often observed in ruminants fed milk replacer [18]. In addition to bioactive proteins, colostrum includes oligosaccharides which probably provide adequate substrates to the pioneer gut bacteria [30]. Limited colostrum intake may then also delay the bacterial colonization of the intestine, possibly leaving the newborn vulnerable to infections during the preweaning period.

### The physiology of the lamb GIT post-weaning was not affected by the pre-weaning rearing mode

To investigate possible long-term effects of the newborn rearing mode on the lamb physiology post-weaning, the GIT anatomy and cellular activity were evaluated in the two groups. No impact of the rearing mode was observed at day 64 on the GIT anatomy as assessed by measuring the relative weight of organs, rumen wall thickness and papillae dimensions. We also quantified the expression of a selection of genes coding for proteins involved in epithelium integrity, metabolism or immune response in the rumen and colon mucosa, but no significant difference could be observed between the ART and MOT lambs. This suggests that after 64 days, the epithelium metabolism and activity is similar whatever the rearing system, even though it could have been different earlier in life. Indeed, the rumen metabolism of pre-weaning lambs has been shown to be mainly stimulated by SCFA [31, 32] and should be then influenced by the activity of the microbiota. In the pre-weaning period, the rumen epithelium undergoes morphological development as well as cellular molecular adaptation of nutrients absorption and metabolism [31, 32]. It was shown that introduction of solid feed to lambs impacts the rumen wall metabolism at 42 days of age [31], hay facilitated establishment of immune function, while concentrate starter enhanced nutrient transport and metabolism. Also in calves, a highly active early microbiome was shown to regulate the rumen development at the cellular level [33]. Anyway, our results suggest that after 64 days of age, the lamb pre-weaning rearing mode had no more impact on the rumen and intestinal epithelium activity, even though significant differences in the rumen microbiota can still be observed between the two groups at day 64 (i.e. *F. succinogenes* population level).

Finally, our data showed differential expression of certain genes in the rumen vs. the colon epithelium of lambs irrespective of the rearing mode and bring additional information to the few papers comparing gene expression in these two GIT segments in sheep. Among the differential genes identified here, *Claudin1*, *HMGSC2, HMGSL* and *MCT1* were more expressed in the rumen wall while *IGF-1, IL-10* and *TLR4*, participating to intestinal homeostasis, epithelial regeneration, and immunity, appeared more expressed in the colon epithelium. Expression of genes involved in innate immune functions has demonstrated variations according to GIT segment in bovines [34]. The expression of *TLR4* (Toll like receptor 4, known to recognize lipopolysaccharide of Gram-negative bacteria) was previously found higher in the colon epithelium than in the rumen mucosa both in calves and cows [34, 35], according to the fact that the colon plays a more important role in the GIT immune system than the rumen. Claudin1 is one of the main tight-junction Claudin proteins which mediate adhesive functions between epithelial cells. Tight junctions play a key role in maintaining the polarity of epithelial cells, regulating the permeability of the epithelial barrier and preventing the translocation of LPS and other toxins [36]. *HMGSC2* and *HMGSL* encode the 3-hydroxy-3-methylglutaryl-CoA synthase isoform 2 and a 3-hydroxy-3-methylglutaryl-CoA lyase, respectively, these two proteins being involved in the ketogenesis pathway. It is known that, while the neonate rumen epithelium is not ketogenic, the ketogenesis pathway is expressed in the sheep rumen mucosa from 42 days of age [37]. MCT1 protein, a monocarboxylate transporter 1 which mediates transport of SCFA across the GIT epithelium, is recognized as highly distributed in the forestomach and large intestine of sheep [38]. More precisely, MCT1 facilitates the efflux of SCFA, lactate and ketone bodies across the basolateral membranes of epithelial cells toward the blood side [38], and its gene expression has been already demonstrated in the ovine rumen epithelium [39]. Our results agree well with MCT1 playing a significant role in the transport of short chain fatty acids and their metabolites in the rumen epithelium of sheep.

### The rearing mode has a strong impact on the rumen and intestinal microbiota of pre-weaned lambs

The present study highlights huge modifications in the rumen and lower gut microbiota colonization depending on the mode of rearing before weaning. Artificial rearing led to lower richness and slower maturation of the rumen microbiota, with delayed establishment of functional fibrolytic populations and protozoa, leading to lower rumen fermentative activities, particularly after weaning.

The rumen and distal gut microbiota of MOT animals diversified and matured with age, according to known stages of GIT colonization in sheep and bovines: Proteobacteria, including facultative anaerobic bacteria, constitute the major phylum during the first days of life, and they consume the oxygen present allowing the gradual colonization of strictly anaerobic bacteria and fungi in the first two weeks [5, 7, 8, 40–43]. From 14 days to weaning, the rumen microbiota grows in complexity, becoming dominated by Bacteroidetes and Firmicutes as a result of a gradual increased in solid feed intake [5, 21, 40]. In the lower gut, as reflected by monitoring the fecal microbiota during the first weeks of age, *Bifidobacterium*, Enterobacteriaceae, *Streptococcus* and *Lactobacillus* taxa decreased while strictly anaerobic bacteria increased and fibrolytic bacteria such as *Ruminococcus flavefaciens* and *Fibrobacter* sp. become detectable only around weaning [44]. Such successions were observed here in MOT rumen and feces, as shown by alpha and beta diversity, microbiota analysis and qPCR quantification, but not in the rumen and feces of ART animals. It has been previously observed that bacterial diversity was higher in mothered lambs compared with lambs fed milk replacer and separated from their dam [19, 20, 22].

The ART rumen and feces microbiota revealed a lactic profile with high proportions of Streptococcaceae and Lactobacillaceae, respectively, compared with MOT microbial communities. This was confirmed by qPCR quantifications which showed higher proportions of lactic acid bacteria and *Bifidobacterium* in the feces of pre-weaned ART lambs. These families/genera are generally associated with primarily milk feeding diet, as it has been shown in humans that milk oligosaccharides and glycans can act as prebiotics stimulating the growth of *Bifidobacterium* and *Lactobacillus* spp [45]. Streptococcaceae may have been promoted by the concentrate intake as it contained quite high levels in starch and sugars. However, it was not possible to measure individually the solid feed intake so the difference in bacterial composition between groups cannot be directly attributed to difference in feed intake. Their higher persistence in ART lamb intestine may reflect the delay in maturation of the rumen and hindgut microbiota and impaired establishment of the fibrolytic communities. Indeed, the cellulolytic bacteria *R. albus* and *F. succinogenes* were already well established at day 7 in the MOT lamb rumens, but, although *R. albus* was also quantified at high levels in ART rumens, *F. succinogenes* establishment was clearly delayed in ART animals. This species was found in lower proportions in the artificially reared lambs, even after weaning. Strong delay in establishment of *F. succinogenes* was previously detected in calves or lambs separated from their dam [5, 10, 46]. A decrease in the Fibrobacteres phylum was also observed at parturition on ewes [47], indicating that *Fibrobacter* may be quite sensitive to changes in the ecological conditions of the rumen. *F. succinogenes* is known as an important fibrolytic microorganism, particularly efficient in degrading recalcitrant cellulosic substrates such as straw [48, 49] and also very sensitive to oxygen.

Another remarkable finding is the much lower relative proportion of Proteobacteria in ART newborn lambs compared with MOT animals, both in rumen (at day 7) and feces (at day 14), the major phylum in ART samples at these ages being Firmicutes. In MOT lambs, the Proteobacteria sequences are in majority affiliated to Neisseriaceae in the rumen and to Enterobacteriaceae including *Escherichia* as dominant genus in the feces. Neisseriaceae are known to colonize the rumen mucosal surface and belong to the core rumen epithelial bacteria of several ruminant species [50, 51]. They were found abundant and active at this site in a metatranscriptome study of the bovine rumen which revealed high expression of genes involved in nitrogen metabolism and of genes related to coping with oxidative stress conditions [52]. Thus, members of this family are assumed to participate to oxygen scavenging, an important function of the rumen epimural microbiota, and could then be very effective in oxygen removal from the MOT rumens, providing conditions suitable to subsequent colonization by strictly anaerobic rumen microbes. Several taxa identified in higher relative abundance in MOT lambs rumen are reported to harbour potential pathogens (e.g. Actinobacteria, Spirochaetes, Tenericutes, [53]); however, MOT lambs didn’t show any clinical sign throughout the entire experimental period.

Because *Escherichia* was a dominant genus in the feces of MOT lambs, we sought the presence of *E. coli* virulence genes in these samples. No difference between MOT and ART lambs’ feces was detected, except for 2 genes with opposite detection frequencies. Despite a strong increase of Enterobacteriaceae relative abundance observed in MOT lambs’ feces at d14, we could not find any strong association with virulence genes detection frequencies of the colibiota, in accordance with the good sanitary status of these animals. Previous studies in piglets have shown that *E. coli* identified during the pre-weaning period were non-pathogenic microorganisms that conferred health benefits to the host [54]. We could thus speculate that in our study the high proportion of *E. coli* might be mostly commensals and that they could fill an ecological niche no more available for pathogenic *E. coli* colonization, potentially providing a protective effect to the mothered lambs.

Among other differential bacterial taxa in the rumen of the two lamb groups, Anaeroplasmataceae and Acidobacteriaceae were much more abundant in MOT rumens. These two families have been associated to the succinate to propionate pathway in goat [55]. They may then be involved in propionate synthesis which favors gluconeogenesis [56].

Regarding the Eukaryota community, artificially reared lambs had no rumen ciliate protozoa at weaning, while natural rearing promoted an early colonization of the rumen by a diverse protozoal population. These results confirm previous findings on lambs and goat kids [10, 22, 23]. The MOT lamb rumen protozoa belonged to many different ciliate taxa, with *Entodinium*, *Eudiplodinium* and *Polyplastron* as dominant genera. It is known that the establishment of ciliate protozoa in the rumen requires the preliminary establishment of a diverse bacteria community [3] so the higher bacterial diversity observed in the rumen of MOT lambs certainly triggered better ecological conditions for ciliate protozoa settlement. Both lamb groups showed a diverse anaerobic fungi community from the Neocallimastigomicota phylum, including monocentric and polycentric taxa, with no difference in composition in the rumens and feces of both groups although colonization by anaerobic fungi was reported to be perturbed during artificial rearing of young ruminants [10, 23]. The ability of anaerobic fungi to form resistant spores may allow them to retain good viability, even in a less anaerobic environment [57]. However, regarding other fungi phyla, MOT feces showed higher relative proportions of Cryptomycota and Chytridiomycota while several yeast genera from the Saccharomycetales order had higher relative abundance in ART rumens and feces. Yeasts such as *Candida* spp. have been found in the sheep farm environment and animal feed, as well as in ewe’s milk and teat surface or ruminant digestive tract [58, 59], but the fungal diversity within farms and according to farming practices has not been studied much. It is then hard to make assumptions about differences found in ART and MOT lambs about these communities.

Finally, the dysbiosis observed in the ART rumen and hindgut microbiota reveals impaired establishments of functionally important microbes, which may be due to several factors. Nutritional factors such as limited colostrum feeding or quality of milk replacer may be important in the artificial rearing management, but other stresses such as the separation with the dam and other adult ruminants may also affect the health and wellbeing of the lamb [12].

## Conclusion

The presence of the mother and rearing mode influence GIT microbiota and health associated parameters in offspring in early life. Although ART and MOT lambs presented similar GIT development and performances, rumen maturation was delayed in ART lambs with very late establishment of functional fibrolytic populations, linked to lower rumen fermentative activities. ART lambs presented also altered sanitary status, probably indicative of higher risk of gut dysbiosis, as reflected by the measured higher LAB fecal concentrations. Higher proportions of commensal *E. coli* populations in the intestine of MOT lambs could exert a protective effect against pathogenic *E. coli*. The first month of age is thus a critical period where the GIT environment and microbiota are particularly unstable and special care should be taken in the management of artificially reared newborn ruminants.

## Methods

### Animals and diet

The animal trial was conducted at the animal facilities of INRAE Herbipôle Experimental Unit UE1414 (10.15454/1.5572318050509348E12 ; Clermont Auvergne Rhône Alpes center). Procedures on animals were carried out in accordance with the guidelines for animal research of the French Ministry of Agriculture and all other applicable national and European guidelines and regulations for experimentation with animals (http://www2.vet-lyon.fr/ens/expa/acc_regl.html). The protocol was validated by the Regional Ethics Committee on Animal Experimentation C2EA-02 and authorized by the French ministry with the reference number 14981-2018050417167566V3.

Fourteen pairs of twin lambs (*Ovis aries*, Romane breed) were used for this study and were followed from birth to 64 days of age (Supplementary Fig S11). Each pair of twins was assigned to two groups: in the first group, lambs were kept with their dam (Mothered - MOT) and in the second group, lambs were separated from their dam after the 1st colostrum intake (usually within 12 h) and artificially-fed with milk replacer (ART). The weight of the lambs at birth were (mean ± SEM) 3.483 ± 0.321 kg and 3.967 ± 0.265 kg for the ART and MOT groups, respectively. The two pens (MOT and ART) were physically separated to ensure no contact between the groups throughout the entire experiment. Bedding was made of straw. ART lambs were bottle fed during the first day, then they were trained to suckle teats (8 teats were proposed per group, in an in-house suckling feeding sytem) until they became enough autonomous. Milk replacer was prepared twice a day by mixing milk powder (Bonilait protéines, Chasseneuil du Poitou, France) with hot water (40 °C maximum). When lambs were 8 days old, they all received a pelleted concentrate (Table S4) until the end of the experiment, in addition to good quality meadow hay and good quality water.

The ewe’s diet postpartum was designed to meet the requirements of the dam in order to feed only one lamb. Each ewe was fed with 600 g of concentrate (Table S5) and 3 kg of meadow hay, covering slightly more than 135% of its energy needs (Table S6).

### Sanitary status assessment

Growth and sanitary status of the lambs were monitored, and rumen contents, feces and blood were sampled up to 55d (Supplementary Fig S11). Growth was monitored by weighing lambs at day 0, 10, 20, 42 and 60 of age. Mortality and sanitary status of the animals were recorded.

The welfare score of the lambs was determined as described previously [12]: it includes several criteria such as nasal and ocular discharge, lameness, perianal dirtiness and diarrhea. Dirtiness of the rear end was assessed visually and given one of the following scores: 0 = without faeces; 1 = sporadic presence of feces; 2 = feces on tail, anus and legs. The other parameters were given a score of 0 if absent or of 1 if present [12]. Notation was performed by the same trained operator, once a week at the same time of the day.

### Sample collection on live animals

The days of sampling are shown on Supplementary Fig S11.

*Rumen and feces sampling*. Rumen contents were collected via oesophageal tubing as previously described [10] using a PVC tube whose diameter was adapted to the lamb age and morphology. Contention of lambs was smooth, and the tube was carefully introduced in the mouth of the lamb and pushed gently inside the rumen. The regurgitated digestive contents were retrieved in a sterile tube. The quality of the sample was visually checked (absence of milk, absence of saliva, no trace of blood). During the milk feeding period, lambs were sampled in the morning after the milk meal while when lambs consumed solid feed, animals were sampled before the morning feeding. Immediately after sampling, pH was recorded, and samples were brought back to the laboratory where they were processed.

The sample was then divided in three sub-samples: one was treated for analysis of short chain fatty acids (SCFA), one for protozoa enumeration, and the other was frozen at − 20 °C until microbiota analysis with qPCR and sequencing.

Fecal contents were collected manually in the rectum of lambs and frozen at − 20 °C for further analysis.

*Blood sampling.* Blood samples were collected from all lambs at day 2, 7, 28 and 55 of age through the jugular vein in dry collection tubes (Becton Dickinson, Franklin Lakes, NJ, USA) by a qualified technician. Dry tubes were set for clotting for at least 1 h at room temperature, centrifuged at 4 500 rpm for 20 min at 4 °C and serum supernatants were stored at -80 °C until further analysis.

### Microbiota analysis

Protozoa were enumerated in a Neubauer chamber under a microscope after staining with methyl green solution as previously described [10].

DNA was extracted from at least 250 mg of rumen or fecal content using the Quick DNA Fecal/Soil Microbe kit (Zymo Research, Irvine, CA, USA). DNA yield and quality were determined after Nanodrop 1000 and Qubit spectrophotometric quantifications. DNA extracts were stored at -20 °C until analysis.

#### qPCR quantification of microbial populations and specific E. coli virulence genes

Microbial populations were quantified using qPCR method, with specific primer sets and PCR conditions targeting ribosomal RNA genes of total bacteria, specific bacterial groups, genus, or species according to the digestive sample (rumen fluid or feces). PCR targets and primers are summarized in Supplementary Table S7. Selected genes associated to *E. coli* virulence were also quantified in feces samples through the same qPCR method (Supplementary Table S8). Standards were used to determine the absolute abundance of each target, expressed as the Log_10_ number of gene copies per microgram of pelleted rumen or feces. For total bacteria and cellulolytic bacteria, the standard curves were prepared according to Mosoni et al. (60) using the DNA extracted from reference strains indicated in the Supplementary Table S6.

#### Metataxonomic analysis

Microbiota diversity and taxonomic composition were analysed by 16S/18S rRNA gene amplicon sequencing. DNA samples were quantified with a Qubit spectrophotometer to adjust concentrations to at least 20 ng/µL, and a volume of 30 µL per sample was sent to the Novogene sequencing platform (Novogene Co. Ltd, UK). DNA sequencing was performed on a subset of samples from 8 lambs per group for each sampling time considered. The diversity and composition of rumen/fecal microbiota were studied using the high throughput sequencing Illumina MiSeq (Illumina, San Diego, CA) method (2 x 250 nt paired ends). The primer sets used, and rDNA regions targeted are indicated in Supplementary Table S5. Libraries construction and MiSeq Illumina sequencing were carried out following protocols validated by Novogene. Bioinformatic analysis on alpha and beta diversity indices as well as taxonomic assignment were performed by Novogene for eukaryotes.

Bacterial taxonomic assignment was performed using the rANOMALY [61] pipeline based on the DADA2 package and has been used on R 3.5.1 (R Core Team (2021). R: A language and environment for statistical computing. R Foundation for Statistical Computing, Vienna, Austria. URL https://www.R-project.org/) for the pipeline’s step of filtering, trimming, dereplication, to infer the sample composition and to remove chimera. The multiple-sequence alignment was performed using the DECIPHER R package [62]. The Silva nr v.132 database was used to assign taxonomy from Kingdom to Genus. Stringent ASV filtering was applied according to Husso et al., [63].

All the diversity analyses were performed with the rANOMALY [61] pipeline based on the Phyloseq R package [64]. Raw ASV abundances were used for the alpha-diversity analyses. Beta diversity analyses were performed using transformed abundance table with the DESeq2’s variance stabilizing transformation [65]. The overall dissimilarity of the microbial community between ages and groups was evaluated by principal coordinates analysis (PCoA) based on Bray-Curtis dissimilarity. The significance of differences between groups was tested by analysis of similarity (ANOVA). The microbiome differential abundance testing and log2foldChange estimate [66] were performed using the default multiple-inference correction of DESeq2 (Benjamini-Hochberg). Indicator species in rumen and in feces were identified using the “indicspecies” package in R [67, 68]. This method identify species that are specific to one group with high fidelity (most samples in that group have the species). For this, a specie-level identity ASV table was used as input. Each specie ecological niche preference (ART or MOT at each sampling days) was identified using the Pearson’s phi coefficient of association (corrected for unequal sample sizes) using the “indicspecies” package and 10,000 permutations. All samples were considered as independent.

### SCFA and IgG quantification

The serum Immunoglobulin G (IgG) concentration was analysed by radial immunodiffusion by CIAL Sud Ouest laboratory (Auch, France) from 1 ml lamb serum sample.

SCFA concentrations were determined in rumen samples by gas chromatography, as previously described [10].

### Organ and tissue collection at slaughter

At day 64, 5 lambs from each group were slaughtered in the experimental slaughterhouse of the INRAE Herbipôle facility (Saint-Genès-Champanelle, France, Permit number: 63,345,001).

The different organs of the GIT tract were collected just after slaughter. The organ contents were withdrawn, and the organs were rinsed with tap water and weighted.

Morphological measures were performed on the digestive organs, and rumen and intestinal biopsies were collected for transcriptomic study of targeted genes using RT-qPCR (Supplementary Table S9).

The rumen tissue was taken from the ventral sac which was predominately exposed to rumen liquor prior to slaughter. The colon was unrolled, and tissue was collected from the medium part.

The collected rumen and colon tissues were rinsed with PBS 1X at ambient temperature, followed by rinsing in ice-cold physiological saline. Tissues were then cut with sterile scalpels into 4–5 mm^2^ fragments, put into sterile Eppendorf tubes and flash frozen in liquid nitrogen before being stored at − 80 °C until RNA extraction.

### RNA extraction from tissues and gene expression analysis

Total RNA was extracted from rumen and colon samples using Trizol (Invitrogen) followed by DNase digestion and Qiagen RNeasy column purification (Qiagen). The nucleic acid concentration and purity.

were assessed using a NanoDrop spectrophotometer (ThermoFisher Scientific). The RNA integrity was finally verified using an Agilent Bioanalyzer 2100 (Agilent, Palo Alto, CA, USA).

The gene expression analysis was performed by Helixio (Saint-Beauzire, France) as described below. Five hundred nanograms of total RNA were reverse transcribed with the “High-Capacity cDNA Reverse Transcription kit” (ThermoFisher Scientific) in a reaction volume of 80 µl, according to the manufacturer recommendations. The samples were incubated at 25 °C for 10 min, followed by 2 h at 37 °C, then the enzyme was inactivated at 85 °C for 5 min. A control was included (no reverse transcriptase) to verify no genomic DNA contamination. The SYBRgreen chemistry was used for qPCR quantification of the gene expression. The reaction mix contained 1X PowerUp™ SYBR™ Green Master Mix (2X) (ThermoFisher Scientific), 500 nM of forward and reverse primers (Table S6), and 10 ng of cDNA in a reaction volume of 20 µl. qPCR amplification were performed in triplicate and according to ThermoFisher Scientific instructions using a TaqMan 7900 Fast instrument (ThermoFisher Scientific). A no template control was included for each gene. The relative expression of the targeted genes was calculated using the comparative Ct method (2^−ΔΔ*C*^_T_ method) [69] and two housekeeping genes as references (β-actin ACTB and glyceraldehyde-3-phosphate dehydrogenase GAPDH). These genes have been widely used as housekeeping genes in various cellular models [70].

### Statistical analysis

Graphical representations and statistical analyses were performed using GraphPad Prism 9.0.2. The effect of rearing mode (ART or MOT) and Time factors and their interaction were evaluated using a linear mixed model with repetitions and multiple comparisons with Sidak’s adjustment.

For some parameters, the data could not fit the chosen mixed model i.e., when the data were not normally distributed and the nonparametric Mann–Whitney (MW) test was thus applied to compare the ART and MOT groups at each time. Due to the sporadic detection of virulence genes, results were analysed as detection frequencies and statistical differences were tested via Fisher exact test. Statistical significance was determined at a p-value < 0.05 and trends were discussed when p < 0.10.

## Electronic supplementary material

Below is the link to the electronic supplementary material.

Supplementary Material 1

Supplementary Material 2

Supplementary Material 3

Supplementary Material 4

## Data Availability

The sequence datasets generated during the current study are available in the SRA repository, (https://www.ncbi.nlm.nih.gov/sra/) as PRJNA882381. All the other data are included in this published article and its supplementary information files.

## References

[1] Arshad MA, Hassan F, ul, Rehman MS, Huws SA, Cheng Y, Din AU. Gut microbiome colonization and development in neonatal ruminants: Strategies, prospects, and opportunities. Animal Nutrition. 2021 Sep 1;7(3):883–95. 10.1016/j.aninu.2021.03.004 PMC8484983 34632119

[2] Yao Y, Cai X, Ye Y, Wang F, Chen F, Zheng C (2021). The role of Microbiota in Infant Health: from early life to Adulthood. Front Immunol.

[3] Fonty G, Senaud J, Jouany JP, Gouet P. Establishment of ciliate protozoa in the rumen of conventional and conventionalized lambs: influence of diet and management conditions. Can J Microbiol. 1988 Mar;34(3):235–41. 10.1139/m88-044 3138013

[4] Fonty G, Jouany JP, Chavarot M, Bonnemoy F, Gouet P (1991). Development of the rumen digestive functions in lambs placed in a sterile isolator a few days after birth. Reprod Nutr Dev.

[5] Jami E, Israel A, Kotser A, Mizrahi I. Exploring the bovine rumen bacterial community from birth to adulthood. ISME J. 2013 Jun;7(6):1069–79. 10.1038/ismej.2013.2 PMC3660679 23426008

[6] Rey M, Enjalbert F, Monteils V. Establishment of ruminal enzyme activities and fermentation capacity in dairy calves from birth through weaning. J Dairy Sci. 2012 Mar;95(3):1500–12. 10.3168/jds.2011-4902 22365231

[7] Rey M, Enjalbert F, Combes S, Cauquil L, Bouchez O, Monteils V. Establishment of ruminal bacterial community in dairy calves from birth to weaning is sequential. J Appl Microbiol. 2014 Feb;116(2):245–57. 10.1111/jam.12405 24279326

[8] Fonty G, Gouet P, Jouany JP, Senaud J (1987). Establishment of the Microflora and anaerobic Fungi in the Rumen of lambs. Microbiology.

[9] Meale SJ, Chaucheyras-Durand F, Berends H, Guan LL, Steele MA. From pre- to postweaning: Transformation of the young calf’s gastrointestinal tract1. J Dairy Sci. 2017 Jul;100(1):5984–95. 10.3168/jds.2016-12474 28527800

[10] Chaucheyras-Durand F, Ameilbonne A, Auffret P, Bernard M, Mialon MM, Dunière L, et al. Supplementation of live yeast based feed additive in early life promotes rumen microbial colonization and fibrolytic potential in lambs. Sci Rep. 2019 Dec;16(1):19216. 10.1038/s41598-019-55825-0 PMC6914811 31844130

[11] Gaudin S, Chaillou E, Wycke MA, Cornilleau F, Moussu C, Calandreau L (2018). All bonds are not alike: a psychoendocrine evaluation of infant attachment. Dev Psychobiol.

[12] Mialon MM, Boivin X, Durand D, Boissy A, Delval E, Bage AS, et al. Short- and mid-term effects on performance, health and qualitative behavioural assessment of Romane lambs in different milk feeding conditions. Animal. 2021 Mar;15(3):100157. 10.1016/j.animal.2020.100157 33454276

[13] David I, Bouvier F, Ricard E, Ruesche J, Weisbecker JL. Feeding behaviour of artificially reared Romane lambs. Animal. 2014 Jan;8(1):982–90. 10.1017/S1751731114000603 PMC4023569 24666599

[14] Hernández-Castellano LE, Almeida AM, Castro N, Argüello A. The colostrum proteome, ruminant nutrition and immunity: a review. Curr Protein Pept Sci. 2014 Feb;15(1):64–74. 10.2174/1389203715666140221124622 24555887

[15] Ahmad R, Khan A, Javed MT, Hussain I. The level of immunoglobulins in relation to neonatal lamb mortality in Pak-Karakul sheep. Vet arhiv. 2000;11.

[16] Hodgson JC, Moon GM, Hay LA, Quirie M. Effectiveness of substitute colostrum in preventing disease in newborn lambs. BSAP Occasional Publication. 1992 ed;15:163–5.

[17] Vihan VS. Immunoglobulin levels and their effect on neonatal survival in sheep and goats. Small Ruminant Research. 1988 Jun;1(2):135–44.

[18] Lora I, Gottardo F, Bonfanti L, Stefani AL, Soranzo E, Dall’Ava B, et al. Transfer of passive immunity in dairy calves: the effectiveness of providing a supplementary colostrum meal in addition to nursing from the dam. Animal. 2019 Nov;13(11):2621–9. 10.1017/S1751731119000879 31062681

[19] Belanche A, Yáñez-Ruiz DR, Detheridge AP, Griffith GW, Kingston-Smith AH, Newbold CJ (2019). Maternal versus artificial rearing shapes the rumen microbiome having minor long-term physiological implications. Environ Microbiol.

[20] Abecia L, Ramos-Morales E, Martínez-Fernandez G, Arco A, Martín-García AI, Newbold CJ, et al. Feeding management in early life influences microbial colonisation and fermentation in the rumen of newborn goat kids. Anim Prod Sci. 2014 Jul;23(9):1449–54.

[21] Yáñez-Ruiz DR, Abecia L, Newbold CJ (2015). Manipulating rumen microbiome and fermentation through interventions during early life: a review. Front Microbiol.

[22] Belanche A, Cooke J, Jones E, Worgan HJ, Newbold CJ. Short- and long-term effects of conventional and artificial rearing strategies on the health and performance of growing lambs. Animal. 2019 Apr;13(4):740–9. 10.1017/S1751731118002100 30117410

[23] Palma-Hidalgo JM, Jiménez E, Popova M, Morgavi DP, Martín-García AI, Yáñez-Ruiz DR, et al. Inoculation with rumen fluid in early life accelerates the rumen microbial development and favours the weaning process in goats. Anim Microbiome. 2021 Jan;19(1):11. 10.1186/s42523-021-00073-9 PMC7814744 33499992

[24] Furman O, Shenhav L, Sasson G, Kokou F, Honig H, Jacoby S et al. Stochasticity constrained by deterministic effects of diet and age drive rumen microbiome assembly dynamics. Nat Commun 2020 Apr 20;11(1):1904. 10.1038/s41467-020-15652-8 PMC7170844 32312972

[25] Santin M. Cryptosporidium and Giardia in Ruminants. Vet Clin North am Food Anim Pract. 2020 Mar;36(1):223–38. 10.1016/j.cvfa.2019.11.005 32029186

[26] Keeton STN, Navarre CB. Coccidiosis in large and small ruminants. Vet Clin North am Food Anim Pract. 2018 Mar;34(1):201–8. 10.1016/j.cvfa.2017.10.009 29249601

[27] Alves AC, Alves NG, Ascari IJ, Junqueira FB, Coutinho AS, Lima RR, et al. Colostrum composition of Santa Inês sheep and passive transfer of immunity to lambs. J Dairy Sci. 2015 Jun;98(6):3706–16. 10.3168/jds.2014-7992 25828655

[28] Hammon HM, Liermann W, Frieten D, Koch C, Review. Importance of colostrum supply and milk feeding intensity on gastrointestinal and systemic development in calves. Animal. 2020 Mar;14(S1):133–43. 10.1017/S1751731119003148 32024575

[29] Fischer AJ, Song Y, He Z, Haines DM, Guan LL, Steele MA. Effect of delaying colostrum feeding on passive transfer and intestinal bacterial colonization in neonatal male holstein calves. J Dairy Sci 2018 Apr;101(4):3099–109. 10.3168/jds.2017-13397 29397179

[30] Dunière L, Renaud JB, Steele MA, Achard CS, Forano E, Chaucheyras-Durand F (2022). A live yeast supplementation to gestating ewes improves bioactive molecule composition in colostrum with no impact on its bacterial composition and beneficially affects immune status of the offspring. J Nutr Sci.

[31] Sun D, Yin Y, Guo C, Liu L, Mao S, Zhu W et al. Transcriptomic analysis reveals the molecular mechanisms of rumen wall morphological and functional development induced by different solid diet introduction in a lamb model. Journal of Animal Science and Biotechnology. 2021 Mar 10;12(1):33. 10.1186/s40104-021-00556-4 PMC7944623 33750470

[32] Liu L, Sun D, Mao S, Zhu W, Liu J. Infusion of sodium butyrate promotes rumen papillae growth and enhances expression of genes related to rumen epithelial VFA uptake and metabolism in neonatal twin lambs. Journal of Animal Science. 2019 Feb 1;97(2):909–21. 10.1093/jas/sky459 PMC6377441 30535158

[33] Malmuthuge N, Liang G, Guan LL. Regulation of rumen development in neonatal ruminants through microbial metagenomes and host transcriptomes. Genome Biology. 2019 Aug 23;20(1):172. 10.1186/s13059-019-1786-0 PMC6708143 31443695

[34] Malmuthuge N, Li M, Fries P, Griebel PJ, Guan LL. Regional and age dependent changes in gene expression of toll-like receptors and key antimicrobial defence molecules throughout the gastrointestinal tract of dairy calves. Vet Immunol Immunopathol. 2012 Mar;15(1):18–26. 10.1016/j.vetimm.2012.01.010 22321738

[35] Bach A, Guasch I, Elcoso G, Chaucheyras-Durand F, Castex M, Fàbregas F et al. Changes in gene expression in the rumen and colon epithelia during the dry period through lactation of dairy cows and effects of live yeast supplementation. Journal of Dairy Science. 2018 Mar 1;101(3):2631–40. 10.3168/jds.2017-13212 29290424

[36] Liu J, Xu T ting, Liu Y, Zhu W, yun, Mao S. yong. A high-grain diet causes massive disruption of ruminal epithelial tight junctions in goats. Am J Physiol Regul Integr Comp Physiol. 2013 Aug 1;305(3):R232-241. 10.1152/ajpregu.00068.2013 23739344

[37] Lane MA, Baldwin RLIV, Jesse BW. Developmental changes in ketogenic enzyme gene expression during sheep rumen development1. Journal of Animal Science. 2002 Jun 1;80(6):1538–44. 10.2527/2002.8061538x 12078735

[38] Kirat D, Inoue H, Iwano H, Hirayama K, Yokota H, Taniyama H et al. Monocarboxylate transporter 1 gene expression in the ovine gastrointestinal tract. The Veterinary Journal. 2006 May 1;171(3):462–7. 10.1016/j.tvjl.2004.12.009 16624712

[39] Connor EE, Li RW, Baldwin RL, Li C. Gene expression in the digestive tissues of ruminants and their relationships with feeding and digestive processes. animal. 2010 Jul;4(7):993–1007. 10.1017/S1751731109991285 22444605

[40] Wang L, Zhang K, Zhang C, Feng Y, Zhang X, Wang X, et al. Dynamics and stabilization of the rumen microbiome in yearling tibetan sheep. Sci Rep. 2019 Dec;23:9:19620. 10.1038/s41598-019-56206-3 PMC6927978 31873173

[41] Klein-Jöbstl D, Schornsteiner E, Mann E, Wagner M, Drillich M, Schmitz-Esser S. Pyrosequencing reveals diverse fecal microbiota in Simmental calves during early development. Frontiers in Microbiology [Internet]. 2014 [cited 2022 Feb 2];5. Available from: https://www.frontiersin.org/article/10.3389/fmicb.2014.00622. 10.3389/fmicb.2014.00622 PMC4233928 25452753

[42] Oikonomou G, Teixeira AGV, Foditsch C, Bicalho ML, Machado VS, Bicalho RC. Fecal microbial diversity in pre-weaned dairy calves as described by pyrosequencing of metagenomic 16S rDNA. Associations of Faecalibacterium Species with Health and Growth. PLoS ONE. 2013 Apr;30(4):e63157. 10.1371/journal.pone.0063157 PMC3639981 23646192

[43] Dill-McFarland KA, Breaker JD, Suen G. Microbial succession in the gastrointestinal tract of dairy cows from 2 weeks to first lactation. Sci Rep. 2017 Jan;18:7:40864. 10.1038/srep40864 PMC5241668 28098248

[44] Uyeno Y, Sekiguchi Y, Kamagata Y (2010). rRNA-based analysis to monitor succession of faecal bacterial communities in Holstein calves. Lett Appl Microbiol.

[45] Milani C, Duranti S, Bottacini F, Casey E, Turroni F, Mahony J et al. The First Microbial Colonizers of the human gut: composition, activities, and Health Implications of the infant gut microbiota. Microbiol Mol Biol Rev 2017 Nov 8;81(4):e00036–17. 10.1128/MMBR.00036-17 PMC5706746 29118049

[46] Yang B, Le J, Wu P, Liu J, Guan LL, Wang J (2018). Alfalfa intervention alters Rumen Microbial Community Development in Hu lambs during early life. Front Microbiol.

[47] Dunière L, Esparteiro D, Lebbaoui Y, Ruiz P, Bernard M, Thomas A et al. Changes in Digestive Microbiota, Rumen Fermentations and oxidative stress around Parturition are alleviated by live yeast feed supplementation to Gestating Ewes. J Fungi (Basel). 2021 Jun 4;7(6):447. 10.3390/jof7060447 PMC8228133 34199914

[48] Neumann AP, Suen G. The phylogenomic diversity of Herbivore-Associated Fibrobacter spp. Is correlated to lignocellulose-degrading potential. mSphere 3(6):e00593–18. 10.1128/mSphere.00593-18 PMC6291624 30541780

[49] Matulova M, Nouaille R, Capek P, Péan M, Forano E, Delort AM. Degradation of wheat straw by Fibrobacter succinogenes S85: a liquid- and Solid-State Nuclear magnetic resonance study. Appl Environ Microbiol. 2005 Mar;71(3):1247–53. 10.1128/AEM.71.3.1247-1253.2005 PMC1065164 15746325

[50] Tan RSG, Zhou M, Li F, Guan LL. Identifying active rumen epithelial associated bacteria and archaea in beef cattle divergent in feed efficiency using total RNA-seq. Current Research in Microbial Sciences. 2021 Dec 1;2:100064. 10.1016/j.crmicr.2021.100064 PMC8610342 34841354

[51] Anderson CJ, Koester LR, Schmitz-Esser S (2021). Rumen Epithelial Communities share a core bacterial microbiota: a Meta-analysis of 16S rRNA gene Illumina MiSeq sequencing datasets. Front Microbiol.

[52] Mann E, Wetzels SU, Wagner M, Zebeli Q, Schmitz-Esser S (2018). Metatranscriptome sequencing reveals insights into the Gene expression and functional potential of Rumen Wall Bacteria. Front Microbiol.

[53] Prescott JF. 22 - Actinomyces, Nocardia, Streptomyces, Dermatophilus, and Rhodococcus. In: Carter GR, Cole JR, editors. Diagnostic Procedure in Veterinary Bacteriology and Mycology (Fifth Edition) [Internet]. San Diego: Academic Press; 1990 [cited 2023 Feb 9]. p. 271–85. Available from: https://www.sciencedirect.com/science/article/pii/B9780121617752500268.

[54] Yang Q, Huang X, Wang P, Yan Z, Sun W, Zhao S, et al. Longitudinal development of the gut microbiota in healthy and diarrheic piglets induced by age-related dietary changes. Microbiologyopen. 2019 Dec;8(12):e923. 10.1002/mbo3.923 PMC6925166 31496126

[55] Denman SE, Martinez Fernandez G, Shinkai T, Mitsumori M, McSweeney CS (2015). Metagenomic analysis of the rumen microbial community following inhibition of methane formation by a halogenated methane analog. Front Microbiol.

[56] Aschenbach JR, Kristensen NB, Donkin SS, Hammon HM, Penner GB (2010). Gluconeogenesis in dairy cows: the secret of making sweet milk from sour dough. IUBMB Life.

[57] McGranaghan P, Davies JC, Griffith GW, Davies DR, Theodorou MK. The survival of anaerobic fungi in cattle faeces. FEMS Microbiology Ecology. 1999 Jul 1;29(3):293–300.

[58] Quintana ÁR, Perea JM, García-Béjar B, Jiménez L, Garzón A, Arias R. Dominant Yeast Community in Raw Sheep’s Milk and Potential Transfers of Yeast Species in Relation to Farming Practices. Animals. 2020 May;10(5):906. 10.3390/ani10050906 PMC7278492 32456104

[59] Fernandes T, Carvalho BF, Mantovani HC, Schwan RF, Ávila CLS. Identification and characterization of yeasts from bovine rumen for potential use as probiotics. J Appl Microbiol. 2019 Sep;127(3):845–55. 10.1111/jam.14350 31211890

[60] Mosoni P, Chaucheyras-Durand F, Béra-Maillet C, Forano E. Quantification by real-time PCR of cellulolytic bacteria in the rumen of sheep after supplementation of a forage diet with readily fermentable carbohydrates: effect of a yeast additive. J Appl Microbiol. 2007 Dec;103(6):2676–85. 10.1111/j.1365-2672.2007.03517.x 18045448

[61] Theil S, Rifa E, rANOMALY (2021). AmplicoN wOrkflow for Microbial community AnaLYsis. F1000Res.

[62] Wright ES, Using (2016). DECIPHER v2.0 to analyze big biological sequence data in R. R J.

[63] Husso A, Jalanka J, Alipour MJ, Huhti P, Kareskoski M, Pessa-Morikawa T, et al. The composition of the perinatal intestinal microbiota in horse. Sci Rep. 2020 Jan;16(1):441. 10.1038/s41598-019-57003-8 PMC6965133 31949191

[64] McMurdie PJ, Holmes S. Phyloseq: an R Package for Reproducible Interactive Analysis and Graphics of Microbiome Census Data. PLoS ONE. 2013 Apr;22(4):e61217. 10.1371/journal.pone.0061217 PMC3632530 23630581

[65] Love MI, Huber W, Anders S. Moderated estimation of fold change and dispersion for RNA-seq data with DESeq2. Genome Biol. 2014 Dec;5(12):550. 10.1186/s13059-014-0550-8 PMC4302049 25516281

[66] Anders S, Huber W. Differential expression analysis for sequence count data. Genome Biology 2010 Oct 27;11(10):R106. 10.1186/gb-2010-11-10-r106 PMC3218662 20979621

[67] De Cáceres M, Legendre P. Associations between species and groups of sites: indices and statistical inference. Ecology. 2009 Dec;90(12):3566–74. 10.1890/08-1823.1 20120823

[68] Legendre P, De Cáceres M (2013). Beta diversity as the variance of community data: dissimilarity coefficients and partitioning. Ecol Lett.

[69] Livak KJ, Schmittgen TD. Analysis of relative gene expression data using real-time quantitative PCR and the 2(-Delta Delta C(T)) Method. Methods. 2001 Dec;25(4):402–8. 10.1006/meth.2001.1262 11846609

[70] de Kok JB, Roelofs RW, Giesendorf BA, Pennings JL, Waas ET, Feuth T, et al. Normalization of gene expression measurements in tumor tissues: comparison of 13 endogenous control genes. Lab Invest. 2005 Jan;85(1):154–9. 10.1038/labinvest.3700208 15543203

