# Supplementary material for: Effects of rearing mode on gastro-intestinal microbiota and development, immunocompetence, sanitary status and growth performance of lambs from birth to two months of age
Source: Anim Microbiome. 2023 Jul 17;5:34. doi: 10.1186/s42523-023-00255-7 (PMC10353247; doi:10.1186/s42523-023-00255-7)
Supplement: Supplementary file 1 — Supplementary Material 1 [file 42523_2023_255_MOESM1_ESM.docx]

**Table S1.** Bacterial taxa identified through indicspecies analysis in Art and Mot groups at each sampling days in rumen and feces, associated pvalues and relative abundance.

| Group | taxa | p.value | %RA |
| --- | --- | --- | --- |
| 07Art-Rumen | Lactobacillus_ingluviei | 0.0001 | 0.903 |
|  | Lactobacillus_saerimneri | 0.0001 | 0.148 |
|  | Streptococcus_sp._27284-01 | 0.0002 | 0.493 |
|  | Prevotella_sp._HUN102 | 0.0002 | 0.414 |
|  | Escherichia_coli | 0.0023 | 5.181 |
|  | Mannheimia_ruminalis | 0.0047 | 0.058 |
|  | Helicobacter_sp._MIT_01-6451 | 0.0358 | 0.012 |
| 07Mot-Rumen | Bifidobacterium_bifidum | 0.0118 | 0.020 |
|  | Stenotrophomonas_rhizophila | 0.0287 | 0.005 |
| 14Art-Rumen | Neisseria_cinerea | 0.0005 | 0.178 |
|  | Pediococcus_acidilactici | 0.001 | 0.005 |
|  | Treponema_berlinense | 0.0329 | 0.102 |
|  | Streptococcus_entericus | 0.0203 | 0.052 |
| 14Mot-Rumen | Prevotella_brevis | 0.0001 | 0.579 |
|  | Sphingomonas_mali | 0.0001 | 0.151 |
|  | Sphingomonas_wittichii | 0.0001 | 0.019 |
|  | Acidobacteria_bacterium_WX27 | 0.0001 | 0.037 |
|  | Staphylococcus_equorum | 0.0001 | 0.123 |
|  | Prevotella_ruminicola | 0.0016 | 0.081 |
|  | Acidobacteria_bacterium_CB_286306 | 0.0001 | 0.085 |
|  | Treponema_sp._OC1 | 0.0013 | 0.020 |
|  | Treponema_sp._S | 0.0015 | 0.023 |
|  | Lachnospira_multipara | 0.0006 | 0.015 |
|  | Acidovorax_facilis | 0.0011 | 0.009 |
|  | Acidobacteria_bacterium_LP6 | 0.0179 | 0.003 |
|  | Desulfovibrio_piger | 0.0211 | 0.008 |
| 28Art-Rumen | Treponema_sp._SF74 | 0.0016 | 0.003 |
|  | Ruminococcus_albus | 0.0077 | 0.173 |
|  | Alistipes_finegoldii | 0.0156 | 0.038 |
|  | Alistipes_shahii | 0.0451 | 0.005 |
| 28Mot-Rumen | Lachnospiraceae_bacterium_CG70 | 0.0001 | 0.147 |
|  | Ruminococcus_sp._YE281 | 0.0001 | 0.010 |
|  | Anaeroplasma_varium | 0.0002 | 1.498 |
|  | Lachnospiraceae_bacterium_CG34 | 0.0005 | 0.149 |
|  | Lachnospiraceae_bacterium_NK4A144 | 0.0001 | 0.036 |
|  | [Clostridium]_papyrosolvens | 0.0001 | 0.014 |
|  | Butyrivibrio_fibrisolvens | 0.0001 | 0.020 |
|  | Treponema_saccharophilum | 0.0012 | 0.054 |
|  | Prevotella_bryantii | 0.0158 | 0.170 |
|  | Lachnospiraceae_bacterium_NK4A179 | 0.0038 | 0.082 |
|  | [Clostridium]_aminophilum | 0.0047 | 0.015 |
|  | Anaerovibrio_lipolyticus | 0.0042 | 0.011 |
| 14Art-Feces | Lactobacillus_saerimneri | 0.0001 | 0.904 |
|  | Pediococcus_acidilactici | 0.0006 | 2.546 |
|  | Faecalicoccus_pleomorphus | 0.01 | 0.222 |
|  | Lactobacillus_coleohominis | 0.0051 | 0.283 |
|  | Lactobacillus_ingluviei | 0.0005 | 6.570 |
|  | Staphylococcus_equorum | 0.0056 | 0.028 |
| 14Mot-Feces | Acinetobacter_lwoffii | 0.0029 | 0.032 |
|  | Butyricicoccus_pullicaecorum | 0.0037 | 0.209 |
|  | Escherichia_coli | 0.0059 | 67.14 |
|  | Bacteroides_fragilis | 0.0138 | 0.048 |
|  | Stenotrophomonas_koreensis | 0.0414 | 0.004 |
| 28Art-Feces | Anaerostipes_hadrus | 0.0176 | 14.02 |
|  | Bifidobacterium_sp._PG12A | 0.0372 | 0.125 |
| 28Mot-Feces | Ruminococcus_flavefaciens | 0.0001 | 1.318 |
|  | Desulfovibrio_piger | 0.0001 | 0.100 |
|  | Bacteroides_dorei | 0.0001 | 0.210 |
|  | Helicobacter_hepaticus | 0.0003 | 0.048 |
|  | Porphyromonas_sp._UQD_309 | 0.0006 | 0.055 |
|  | Streptococcus_minor | 0.0033 | 0.322 |
|  | Anaerotruncus_sp._MT15 | 0.0002 | 0.081 |
|  | Streptococcus_sp._27284-01 | 0.0005 | 0.020 |
|  | Oscillibacter_sp._1-3 | 0.0004 | 0.023 |
|  | Bacteroides_coprocola | 0.0019 | 0.025 |
|  | Faecalibacterium_prausnitzii | 0.0042 | 0.044 |
|  | [Clostridium]_scindens | 0.0026 | 0.127 |
|  | Streptococcus_entericus | 0.0145 | 0.026 |
|  | Prevotella_sp._HUN102 | 0.0074 | 0.380 |
|  | Rothia_nasimurium | 0.0086 | 0.020 |
|  | Moraxella_oblonga | 0.0198 | 0.049 |
|  | Campylobacter_lanienae | 0.0283 | 0.021 |

**Table S2:** Detection frequency of E. coli virulence genes in feces of ART and MOT lambs at d14 and 28 and associated pvalues.

|  | D14 | | | D28 | | |
| --- | --- | --- | --- | --- | --- | --- |
| virulence gene | ART | MOT | P value | ART | MOT | P value |
| LT | 0 | 2 | 0,199 | 0 | 0 | NA |
| Sta | 11 | 0 | < 0,0001 | 3 | 0 | 0,222 |
| East1 | 0 | 5 | 0,011 | 0 | 0 | NA |
| AggR | 4 | 8 | 0,099 | 1 | 3 | 0,596 |
| HlyA | 8 | 9 | 0,386 | 4 | 5 | >0,999 |
| Stx2 | 0 | 1 | 0,458 | 0 | 0 | NA |
| total lambs | 13 | 11 |  | 14 | 14 |  |

**Table S3**: Descriptive parameters of rumen papillae.

|  | ART | | MOT | |  |
| --- | --- | --- | --- | --- | --- |
| Parameter measured | Mean | SD | Mean | SD | P value |
| Rumen wall thickness (mm) | 4.494 | 0.737 | 5.120 | 0.779 | 0.715 |
| Papillae lenght (mm) | 3.260 | 0.412 | 3.724 | 0.530 | 0.904 |
| Papillae width (mm) | 1.353 | 0.335 | 1.318 | 0.149 | 0.904 |
| Number of papillae/cm^2^ | 53.194 | 8.691 | 58.462 | 11.304 | 0.773 |
| Absorptive index (mm^2^/cm²) | 232.196 | 65.224 | 287.132 | 65.997 | 0.773 |

Absorptive index is calculated as papillae length x width x (number of papillae/cm²). Non parametric Mann-Whitney test was applied to determine statistical significance.

**Table S4:** Composition of the concentrate in the lamb diet

| **Ingredient** | **%** |
| --- | --- |
| Barley | 21.5 |
| Soyabean meal | 18.0 |
| Corn | 16.0 |
| Wheat | 16.0 |
| Wheat bran | 10.0 |
| alfalfa | 6.5 |
| Beet pulp | 5.0 |
| Sugar cane molasses | 3.5 |
| Sodium Carbonate | 3.0 |
| Mineral supplement | 0.5 |
| **Chemical composition** | **g/kg of DM** |
| Starch | 389 |
| Sugars | 66 |
| NDF | 209 |
| ADF | 87 |
| ADL | 17 |
| Crude protein | 188 |

**Table S5**: Composition of the commercial concentrate distributed to the ewes

| **Ingredient** | **g/kg of concentrate** |
| --- | --- |
| Corn gluten feed | 179.7 |
| Rapeseed cake | 150.0 |
| Linseed extruded supplement | 146.3 |
| Wheat bran | 127.0 |
| Barley grain | 100.0 |
| Cereal by products | 62.0 |
| Corn meal | 50.0 |
| Sugarcane molasse | 50.0 |
| Beet pulp | 50.0 |
| Wheat grain | 45.0 |
| Mineral supplements | 40.0 |

DM=dry matter; NDF= neutral detergent fiber; ADF= acid detergent fiber.

**Table S6.** Nutritional composition of the diet offered to the ewes postpartum

| **Good quality meadow hay** | |
| --- | --- |
| Kg/d/ewe | 3 |
| Dry matter | 0.85 |
| Kg/d/ewes (DM) | 2.55 |
| **Concentrate** | |
| Kg/d/ewe | 0.6 |
| Dry matter | 0.875 |
| Kg/d/ewe (DM) | 0.525 |
| Forage / Concentrate (on DM basis) | 83/17 |
| **Daily supply (kg/d/ewe)** | |
| NDF | 1.7412 |
| ADF | 0.9512 |
| Crude protein | 0.3732 |
| Fat (from concentrate) | 0.0263 |
| Starch + sugars (from concentrate) | 0.1617 |

DM=dry matter; NDF= neutral detergent fiber; ADF= acid detergent fiber.

**Table S7.** qPCR primers used for quantification of selected microbial populations and for metataxonomy analysis of rumen and feces samples from ART and MOT lambs.

| Microbial target | Target gene | Primer sequence | Ref | Material used for standard curve |
| --- | --- | --- | --- | --- |
| Total bacteria | 16S rDNA | 5’-AGCAGCCGCGGTAAT-3’  5’-CAGGGTATCTAATCCTGTT-3’ | (1) | Mix of DNA from 11 bacterial strains |
| *Fibrobacter succinogenes* | 16S rDNA | 5’-GTTCGGAATTACTGGGCGTAAA-3’  5’-CGCCTGCCCCTGAACTATC-3’ | (1) | *F.succinogenes* S85 |
| *Ruminococcus albus* | 16S rDNA | 5’-CCCTAAAAGCAGTCTTAGTTCG-3’  5’-CCTCCTTGCGGTTAGAACA-3’ | (2) | *R albus* 7 |
| *Faecalibacterium prausnitzii* | 16S rDNA | F: 5’-GGAGGAAGAAGGTCTTCGG-3’  R: 5’-AATTCCGCCTACCTCTGCACT-3’ | (3) | *F. prausnitzii* DSMZ 17677 |
| *Bifidobacterium* | 16SrDNA | F: 5’-CGGGTGAGTAATGCGTGACC-3’  R:5’-TGATAGGACGCGACCCCA-3’ | (4) | *B. breve ATCC15700* |
| *Lactobacillus/Leuconostoc/Pediococcus* group | 16S rDNA | 5’- CGCCACTGGTGTTCYTCCATATA-3’  5’- AGCAGTagggaatcttcca-3’ | (4) | L. acidophilus DSM 20079 |
| Bacterial 16S | V3-V4 | 341F 5’ – CCTAYGGGRBGCASCAG-3’  806R 5‘ - GGACTACNNGGGTATCTAAT-3’ |  |  |
| Fungal 18S | V4 | 528F 5’ – GCGGTAATTCCAGCTCCAA-3’  706R 5’ – AATCCRAGAATTTCACCTCT-3’ |  |  |

**Table S8**. qPCR primers used for quantification of selected virulence genes of feces samples from ART and MOT lambs.

| **Gene** | **Sequence** | **Program** | **Amplicon size (bp)** | **reference strain** | **Ref** |
| --- | --- | --- | --- | --- | --- |
| *LT* Heat-labile enterotoxin | ATTTACGGCGTTACTATCCTC | 95°C, 30s 58°C, 30s 72°C, 30s | 281 | H10407 | (5) |
|  | TTTTGGTCTCGGTCAGATATG |  |  |  |  |
| Sta Heat-stable enterotoxin a | GCTAATGTTGGCAATTTTTATTTCTGTA | 95°C 30 s 53°C, 30s 72°C 30 s | 190 | H10407 | (6) |
|  | AGGATTACAACAAAGTTCACAGCAGTAA |  |  |  |  |
| *EAST1* EaggEC heat-stable enterotoxin | CCATCAACACAGTATATC | 95°C,30 s 53°C,30 s 72°C,30 s | 111 | EAEC O42 | (7) |
|  | GGTCGCGAGTGACGGCTTTGT |  |  |  |  |
| *aggR*  Fimbrial antigen-specific gene | CTAATTGTACAATCGATGTA  AGAGTCCATCTCTTTGATAAG | 95°C,40 s 55°C,40 s 72°C,40 s | 457 | EAEC O42 | (8) |
| hlyA  Hemolysine A | ACG ATG TGG TTT ATT CTG GA- | 95°C, 30s 52°C, 30s 72°C, 30s | 165 | EDL933 | (6) |
|  | CTT CAC GTG ACC ATA CAT AT- |  |  |  |  |
| Stx1  Shigatoxin 1 | GAAGAGTCCGTGGGATTACG | 95°C, 20s 50°C, 20s 72°C, 20s | 130 | EDL 933 | (9) |
|  | AGCGATGCAGCTATTAATAA |  |  |  |  |
| STX2  Shigatoxin 2 | GTGCCTGTTACTGGGTTTTTCTTC | 95°C,30 s 55°C,30 s 72°C,30 s | 118 | EDL933 | (10) |
|  | AGGGGTCGATATCTCTGTCC |  |  |  |  |

For quantification of these genes, the standard curves were prepared using DNA extracted from enterotoxigenic *E.coli* strain H10407, enteropathogenic *E.coli* strain 2348/69, enteroaggregative *E.coli* strain 042 and from Shiga-toxin producing *E.coli* O157 :H7 EDL 933 strain, that were all available in the MEDiS INRAE laboratory.

**Table S9.** Genes targeted for epithelial cell expression study, primer sequences and annealing temperature for RT-qPCR.

| **Gene Name** | **Gene Symbol** | **Function** | **Forward Primer Sequence** | **Reverse Primer Sequence** | **Reference** | **Tm (°C)** |
| --- | --- | --- | --- | --- | --- | --- |
| Cyclin D1 | CCND1 | Proliferation | CGATAAGCCAGCTAACGGGG | CAGGCTGCCTCCGTCTG | (11) | 60 |
| IGF1 | IGF1 | Proliferation | GCTCTCAACATCTCCCATCTCC | CCCATTGCTTCTGAAGTGCAAA | (11) | 60 |
| MCT1 | MCT1 | Absorption | ATCTACGCGGGATTCTTTGGAT | AAGGTCCATCAGCGTTTCAAAC | (11) | 60 |
| HMGSC2 | HMGCS2 | Metabolism | TACCTGGAGCGAGTGGATGA | GGCGAGTCATCTGGATCTGG | (11) | 60 |
| HMGCL | HMGCL | Metabolism | TCCACGAGACGGACTACAAAA | AGAGGCGGCTCCAAAGATG | (11) | 60 |
| OCLN | OCLN | Integrity | ATCAACCCCGGTGCCGGAAG | GTGGTCTTGCTCTGCCCGCC | (12) | 57 |
| Claudin 1 | CLDN1 | Integrity | GCGCTGCCCCAGTGGAAAGT | GGATCTGCCCGGTGCTCTGC | (13) | 60 |
| TLR2 | TLR2 | Integrity | CTGTGTGCGTCTTCCTCAGA | TCAGGGAGCAGAGTAACCAGA | (14) | 60 |
| TLR4 | TLR4 | Integrity | TCAGAAACCTCCGCTACCTTG | TTCTGAAAAGAGTTGCCTGCC | (13) | 55 |
| IL-1β | IL1B | Cytokine | TGGGAGATGGAAACATCCAG | TTTATTGACTGCACGGGTGC | (13) | 50 |
| IL-10 | IL10 | Cytokine | ACTTTAAGGGTTACCTGGGTTG | GAAAGCGATGACAGCGCCGC | (13) | 57 |
| TNFα | TNFA | Cytokine | AACAGCCCTCTGGTTCAAAC | TCTTGATGGCAGACAGGATG | (13) | 60 |
| GAPDH | GAPDH | Housekeeping | GGGTCATCATCTCTGCACCT | GGTCATAAGTCCCTCCACGA | (11) | 60 |
| β-actin | ACTB | Housekeeping | AGTACTCCGTGTGGATTGGC | ACTCCTGCTTGCTGATCCAC | (15) | 60 |

**References of supplementary data**

1. Bayat AR, Kairenius P, Stefański T, Leskinen H, Comtet-Marre S, Forano E, et al. Effect of camelina oil or live yeasts (Saccharomyces cerevisiae) on ruminal methane production, rumen fermentation, and milk fatty acid composition in lactating cows fed grass silage diets. J Dairy Sci. 1 mai 2015;98(5):3166‑81.

2. Mosoni P, Chaucheyras-Durand F, Béra-Maillet C, Forano E. Quantification by real-time PCR of cellulolytic bacteria in the rumen of sheep after supplementation of a forage diet with readily fermentable carbohydrates: effect of a yeast additive. J Appl Microbiol. déc 2007;103(6):2676‑85.

3. Wang W, Li C, Li F, Wang X, Zhang X, Liu T, et al. Effects of early feeding on the host rumen transcriptome and bacterial diversity in lambs. Sci Rep. 31 août 2016;6(1):1‑14.

4. Furet JP, Firmesse O, Gourmelon M, Bridonneau C, Tap J, Mondot S, et al. Comparative assessment of human and farm animal faecal microbiota using real-time quantitative PCR. FEMS Microbiol Ecol. 2009;68(3):351‑62.

5. Osek J, Gallien P, Truszczyñski M, Protz D. The use of polymerase chain reaction for determination of virulence factors of Escherichia coli strains isolated from pigs in Poland. Comp Immunol Microbiol Infect Dis. 1 juill 1999;22(3):163‑74.

6. Osman KM, Mustafa AM, Elhariri M, Abdelhamed GS. The distribution of Escherichia coli serovars, virulence genes, gene association and combinations and virulence genes encoding serotypes in pathogenic E. coli recovered from diarrhoeic calves, sheep and goat. Transbound Emerg Dis. févr 2013;60(1):69‑78.

7. Osek J. Detection of the enteroaggregative Escherichia coli heat-stable enterotoxin 1 (EAST1) gene and its relationship with fimbrial and enterotoxin markers in E. coli isolates from pigs with diarrhoea. Vet Microbiol. 2 janv 2003;91(1):65‑72.

8. Moyo SJ, Maselle SY, Matee MI, Langeland N, Mylvaganam H. Identification of diarrheagenic Escherichia coli isolated from infants and children in Dar es Salaam, Tanzania. BMC Infect Dis. 9 août 2007;7(1):92.

9. Jackson MP, Newland JW, Holmes RK, O’Brien AD. Nucleotide sequence analysis of the structural genes for Shiga-like toxin I encoded by bacteriophage 933J from Escherichia coli. Microb Pathog. févr 1987;2(2):147‑53.

10. Paton AW, Paton JC, Goldwater PN, Manning PA. Direct detection of Escherichia coli Shiga-like toxin genes in primary fecal cultures by polymerase chain reaction. J Clin Microbiol. nov 1993;31(11):3063‑7.

11. Liu L, Sun D, Mao S, Zhu W, Liu J. Infusion of sodium butyrate promotes rumen papillae growth and enhances expression of genes related to rumen epithelial VFA uptake and metabolism in neonatal twin lambs. J Anim Sci. 1 févr 2019;97(2):909‑21.

12. Bach A, Guasch I, Elcoso G, Chaucheyras-Durand F, Castex M, Fàbregas F, et al. Changes in gene expression in the rumen and colon epithelia during the dry period through lactation of dairy cows and effects of live yeast supplementation. J Dairy Sci. mars 2018;101(3):2631‑40.

13. Malmuthuge N, Li M, Goonewardene LA, Oba M, Guan LL. Effect of calf starter feeding on gut microbial diversity and expression of genes involved in host immune responses and tight junctions in dairy calves during weaning transition. J Dairy Sci. 1 mai 2013;96(5):3189‑200.

14. Liang YS, Li GZ, Li XY, Lü JY, Li FD, Tang DF, et al. Growth performance, rumen fermentation, bacteria composition, and gene expressions involved in intracellular pH regulation of rumen epithelium in finishing Hu lambs differing in residual feed intake phenotype. J Anim Sci. avr 2017;95(4):1727‑38.

15. Die JV, Baldwin RL, Rowland LJ, Li R, Oh S, Li C, et al. Selection of internal reference genes for normalization of reverse transcription quantitative polymerase chain reaction (RT-qPCR) analysis in the rumen epithelium. PLoS ONE [Internet]. 24 févr 2017 [cité 20 oct 2020];12(2). Disponible sur: https://www.ncbi.nlm.nih.gov/pmc/articles/PMC5325532/
