# Supplementary material for: Effects of rearing mode on gastro-intestinal microbiota and development, immunocompetence, sanitary status and growth performance of lambs from birth to two months of age
Source: Anim Microbiome. 2023 Jul 17;5:34. doi: 10.1186/s42523-023-00255-7 (PMC10353247; doi:10.1186/s42523-023-00255-7)
Supplement: Supplementary file 4 — Supplementary Material 4 [file 42523_2023_255_MOESM4_ESM.docx]

**Figure S1**. pH measured on rumen samples taken all along the experiment.

The rumen pH of the artificially reared (ART) and mothered lambs (MOT) are represented by white and grey boxes, respectively. Mixed-effects model (REML) showed effect of age (p<0.001), rearing mode (p<0.05) and interaction between them (p<0.01). Sidak’s multiple comparison test indicated that MOT rumen pH was higher than ART rumen pH at 28 days (p<0.001).

**
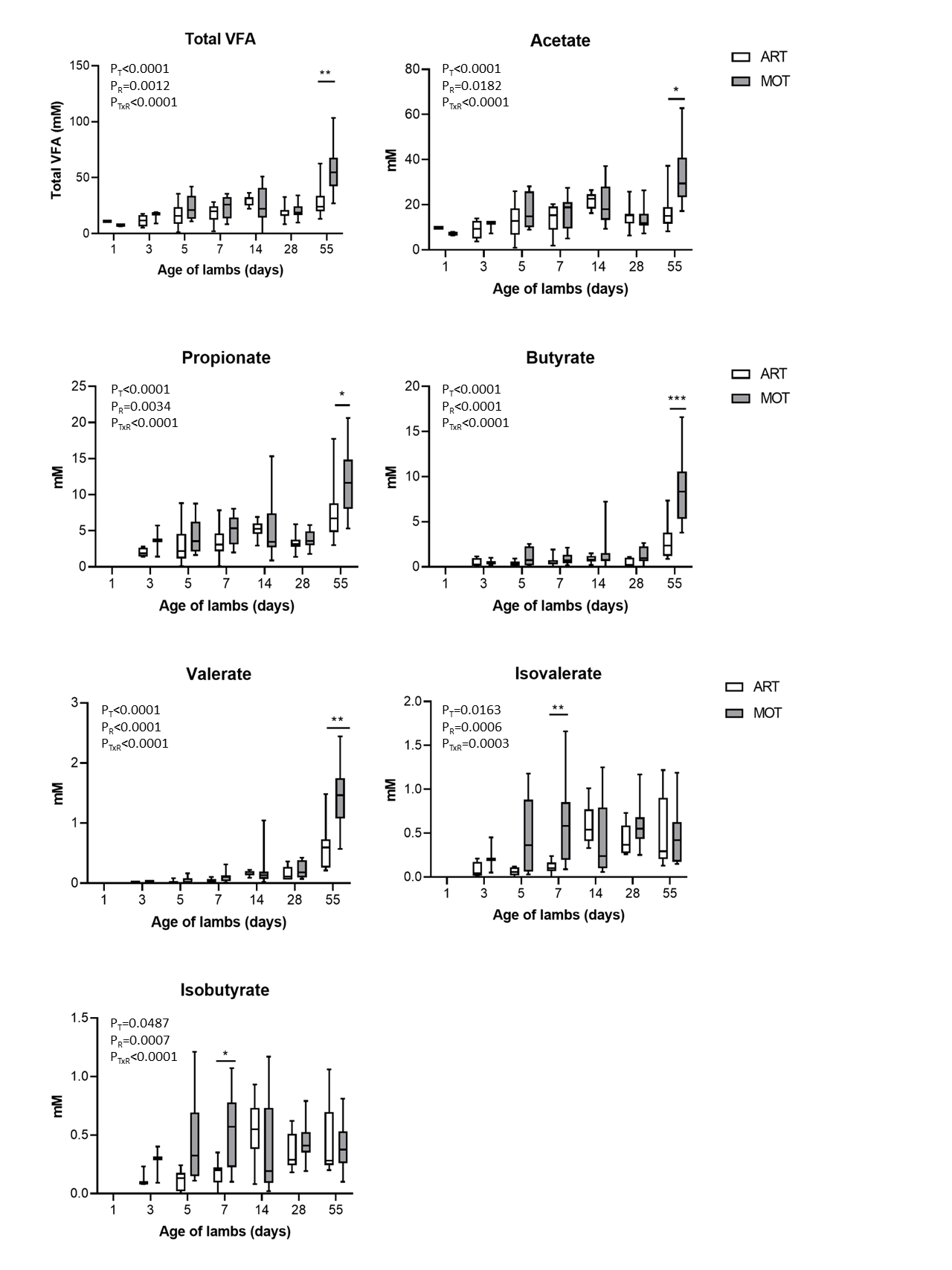
**

**Figure S2.** SCFA concentrations measured in the rumen of ART (white boxes) and MOT (grey boxes)lambs. Mixed-effects model (REML) showed effect of age (p_T_), rearing mode (p_R_) and interaction between them (p_TxR_).

**
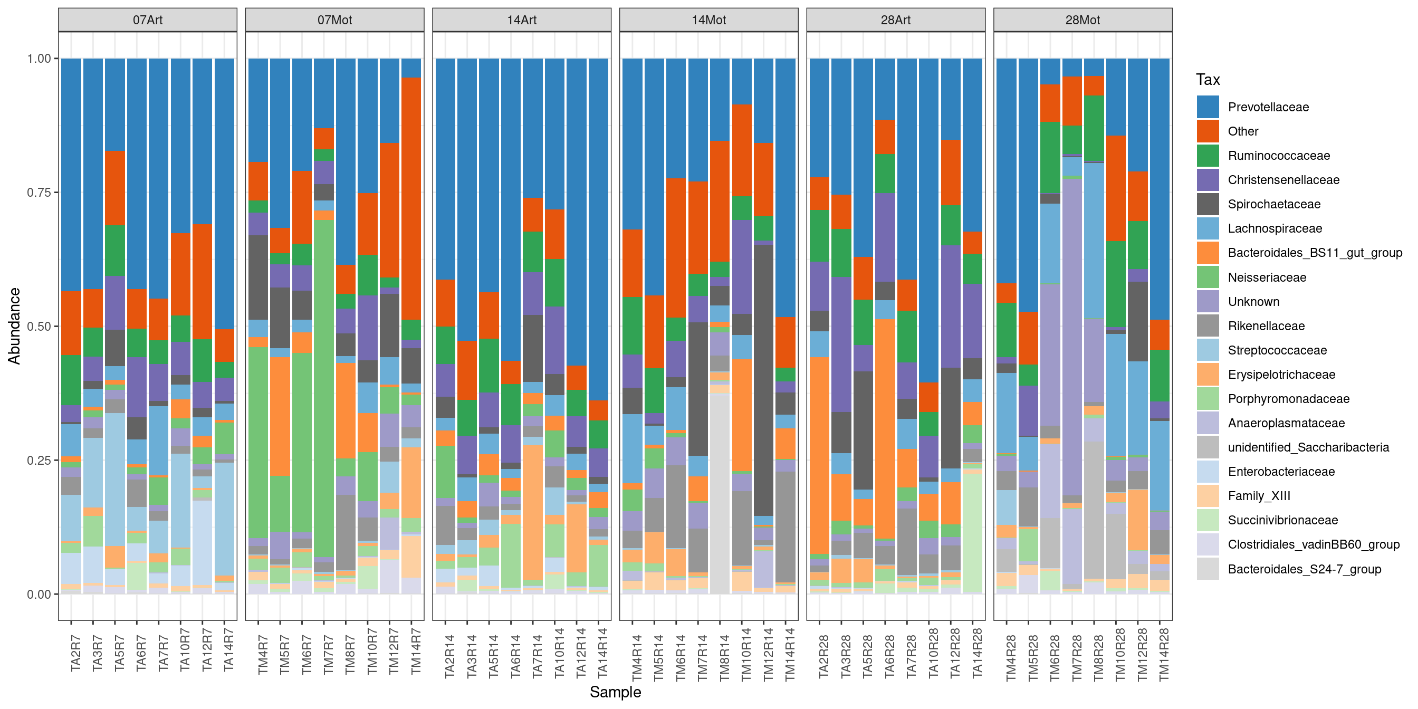
**

**Figure S3**: Composition of the microbiota of the individual rumen samples at the family level.

**
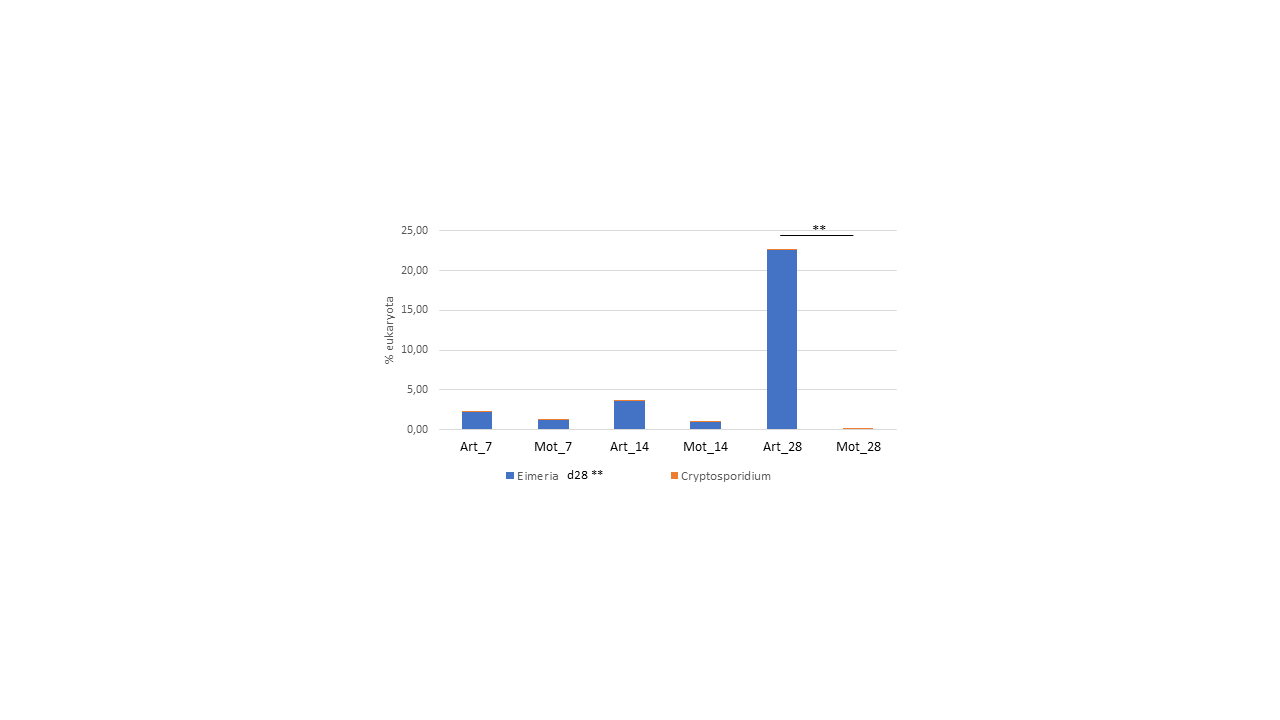
**

**Figure S4**. Parasite taxa identified in the rumen eukaryota community in ART and MOT lamb rumens.


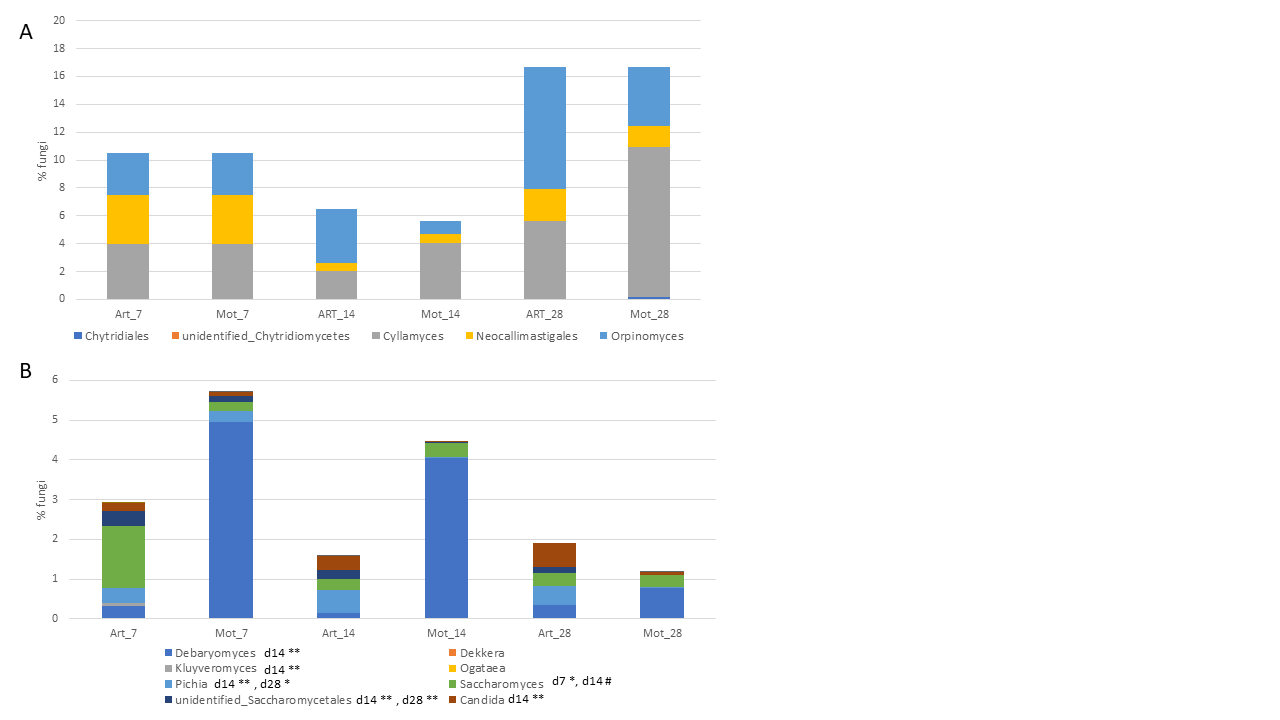


**Figure S5.** Anaerobic fungi (A) and yeast (B) taxa identified the rumen of MOT and ART lambs.


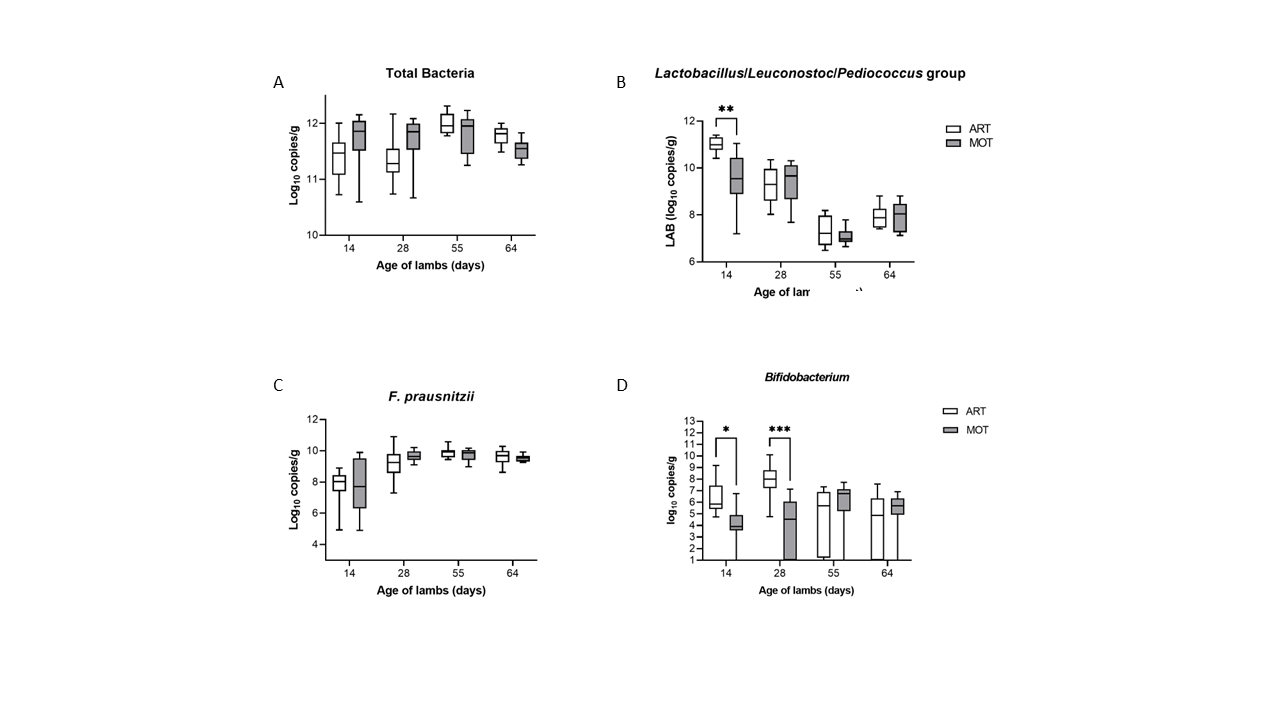


**Figure S6**. qPCR quantification of selected bacterial group in the feces of ART (white boxes) and MOT (grey boxes) lambs. A: total bacteria; B: *Lactobacillus/Leuconostoc/Pediococcus* group; C: *Faecalibacterium prausnitzii*; D: *Bifidobacterium*.


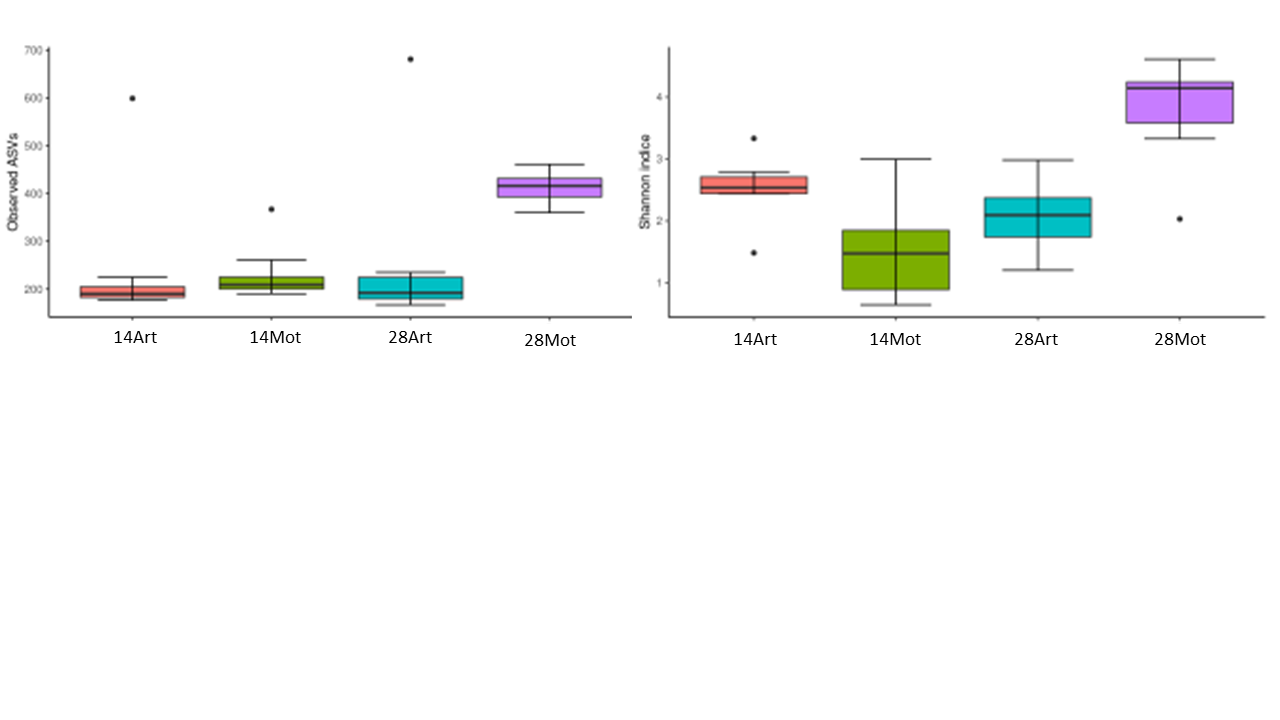


**Figure S7**. Alpha diversity indices of the fecal bacteria populations of the two lamb groups.

**
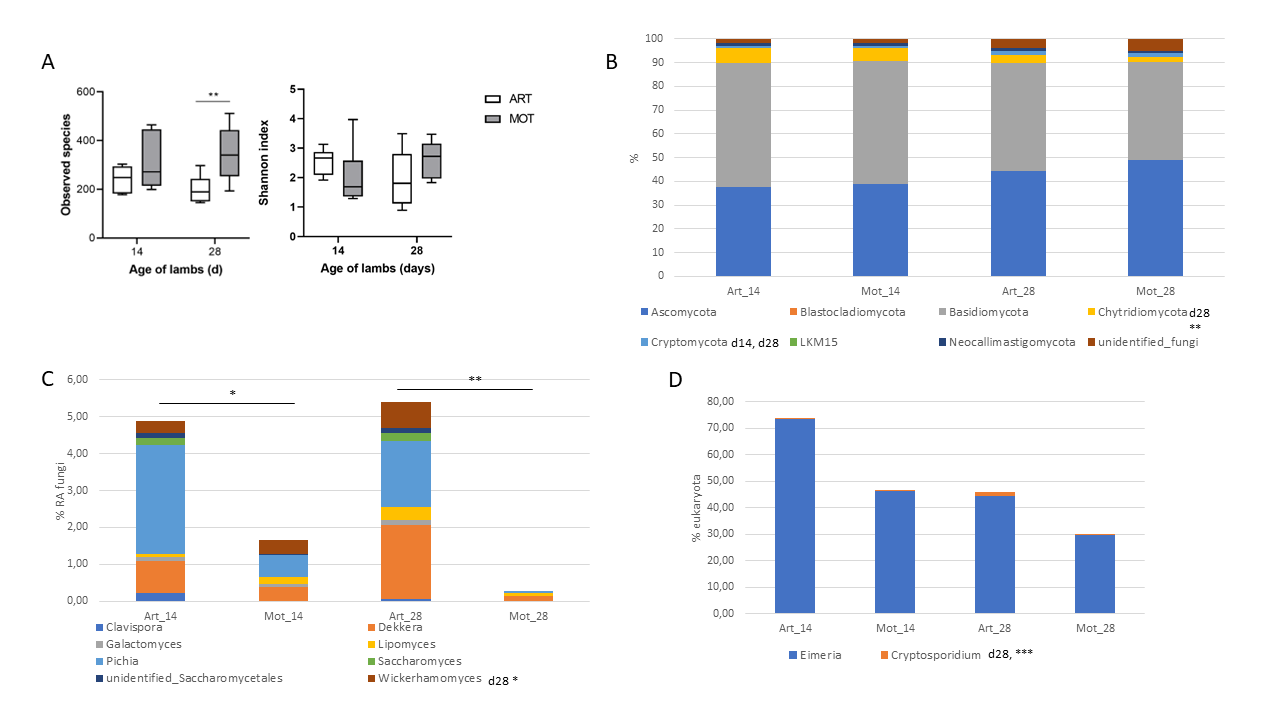
**

**Figure S8.** Effect of rearing mode on fecal eukaryota of the two lamb groups.

A: alpha diversity (observed OTUs and Shannon index) of the fecal eukaryota of ART (white boxes) and MOT (grey boxes) lambs; B,C: composition of the fecal fungi communities (B, phyla; C, Saccharomycetales order); D: identified parasite taxa in the feces of the two groups. Differences between the two groups were analyzed by Mann Whitney non-parametric test.


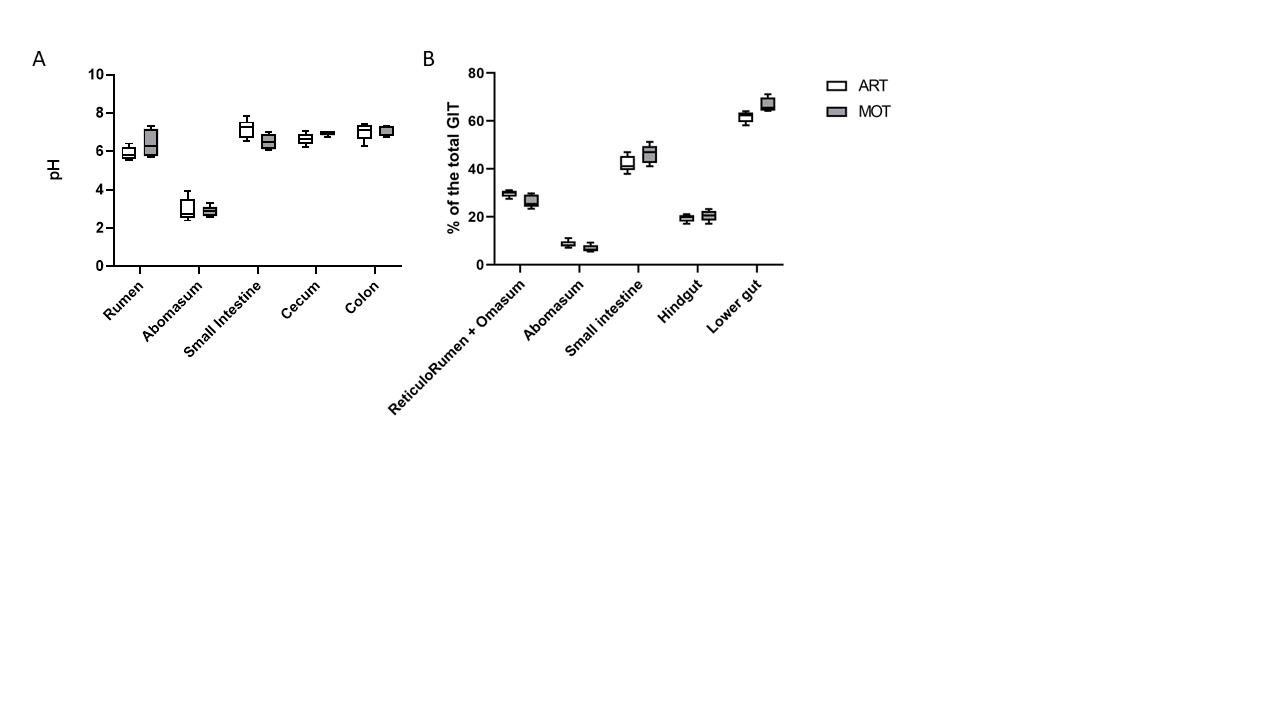


**Figure S9**. pH (A) and relative weight of the digestive tract segments (B) in the ART (white boxes) and MOT (grey boxes) lambs.


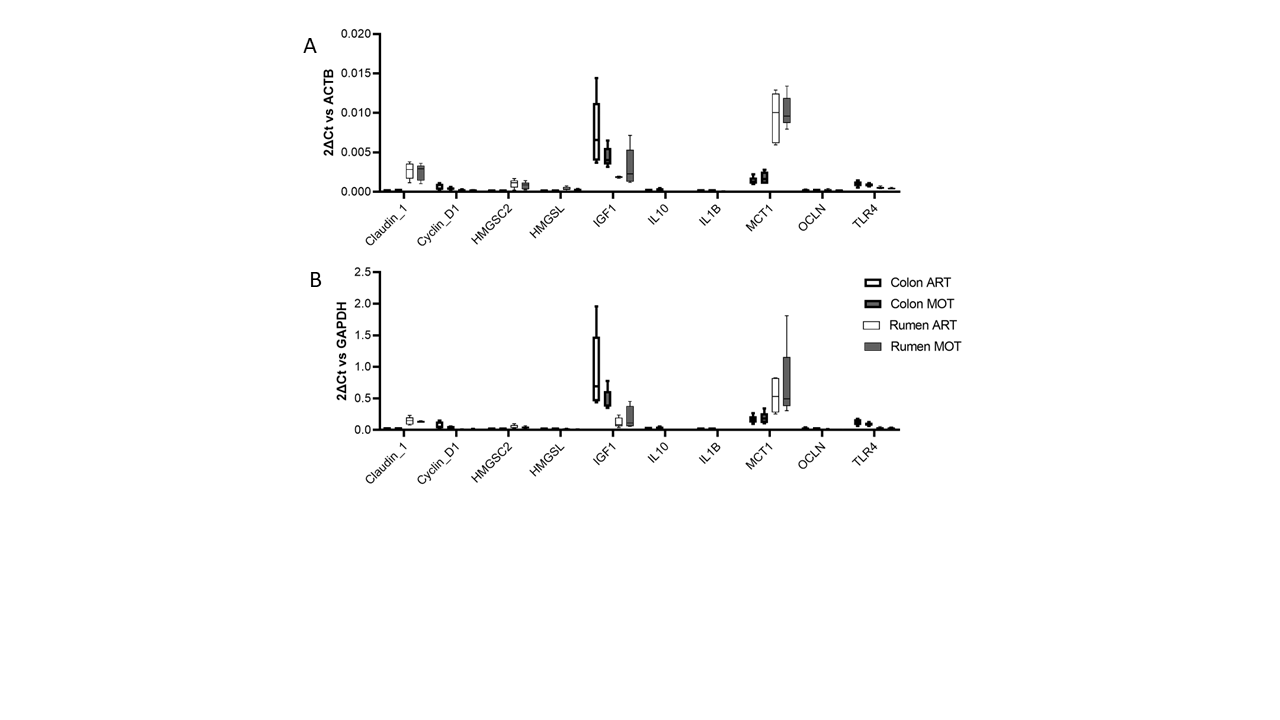


**Figure S10**. Expression of selected genes in the rumen (normal frame) and colon (bold frame) epithelium in ART (white boxes) or MOT (grey boxes) lambs. Gene expression was expressed relative to β-actin gene expression (A) or GAPDH gene expression (B).

**
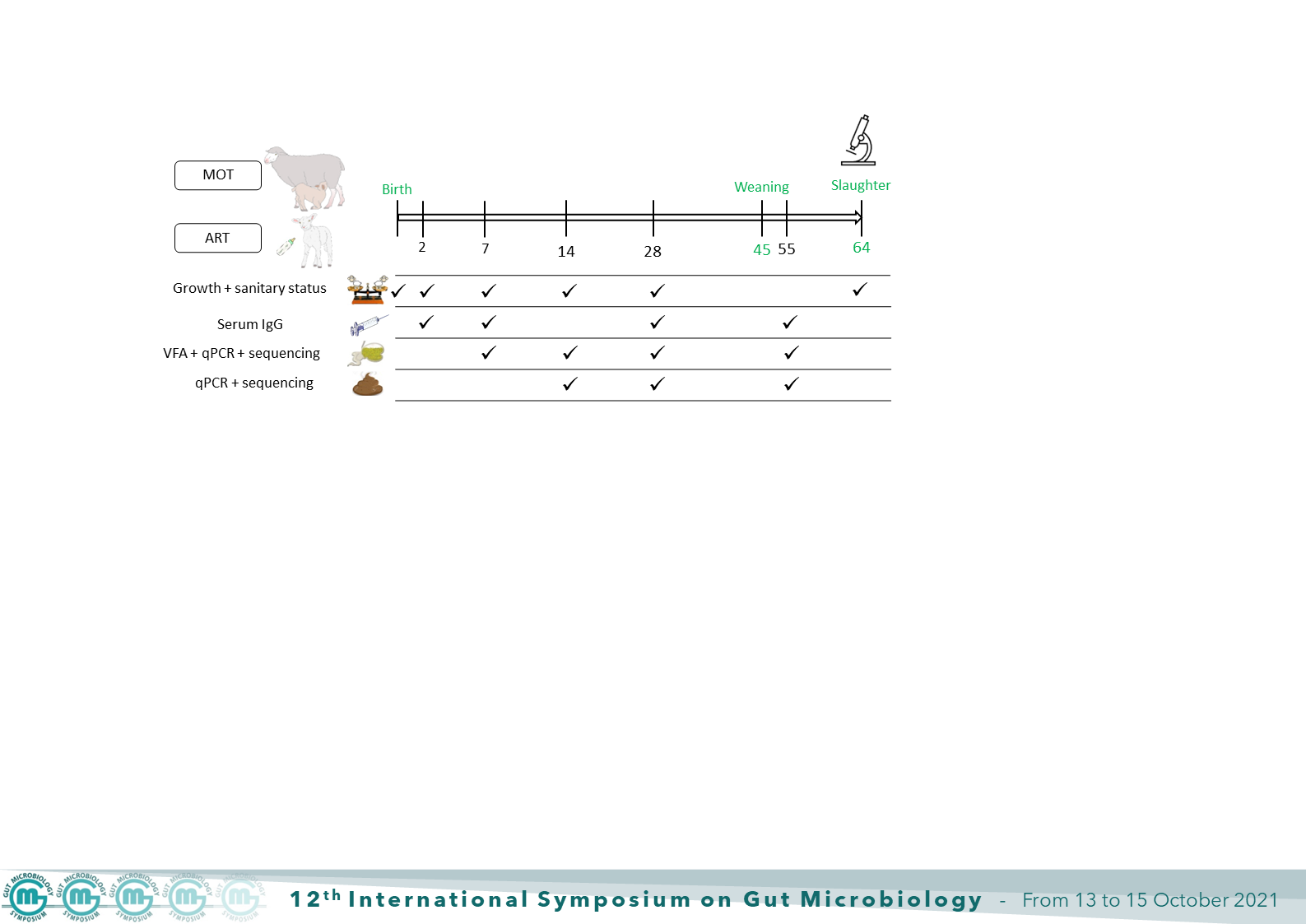
**

**Figure S11**. Experimental design of the animal trial and sampling
